# The Effects of Source Cues and Issue Frames During COVID-19

**DOI:** 10.1017/XPS.2021.3

**Published:** 2021-01-29

**Authors:** Chandler Case, Christopher Eddy, Rahul Hemrajani, Christopher Howell, Daniel Lyons, Yu-Hsien Sung, Elizabeth C. Connors

**Affiliations:** Department of Political Science, University of South Carolina, Columbia SC 29208, USA

**Keywords:** COVID-19, source cues, issue frames, political attitudes

## Abstract

The health and economic outcomes of the COVID-19 pandemic will in part be determined by how effectively experts can communicate information to the public and the degree to which people follow expert recommendation. Using a survey experiment conducted in May 2020 with almost 5,000 respondents, this paper examines the effect of source cues and message frames on perceptions of information credibility in the context of COVID-19. Each health recommendation was framed by expert or nonexpert sources, was fact- or experience-based, and suggested potential gain or loss to test if either the source cue or framing of issues affected responses to the pandemic. We find no evidence that either source cue or message framing influence people’s responses – instead, respondents’ ideological predispositions, media consumption, and age explain much of the variation in survey responses, suggesting that public health messaging may face challenges from growing ideological cleavages in American politics.

## Introduction

The health and economic outcomes of the COVID-19 pandemic are largely dependent on individual behavior – behavior that can potentially be shaped by the information environment. This pandemic thus highlights the importance of communication: how individuals interpret recommendations from experts *matters*, as the effectiveness of this communication can determine how many people are affected by this pandemic and to what extent. Unfortunately, research on the growing distrust of experts and rise of anti-intellectualism in America (Merkley 2020) suggest that communication from experts will not be given the credence it perhaps should be – or worse is downplayed because people do not trust experts.

To attempt to understand individuals’ reception of COVID-19 recommendations, we use a survey experiment and test: (1) whether people’s likelihood of following public health guidelines changes depending on if they originate from an expert or nonexpert source and (2) whether this is moderated by issue frame (experience vs. fact and loss vs. gain). Our findings suggest that neither source cues nor issue frames influence the adoption of information about COVID-19. This is in line with previous literature that suggests highly salient issues are most likely to be subject to motivated reasoning (Jerit and Barabas 2012; Slothuus and de Vreese 2010). Indeed, we find that individuals’ responses are largely driven by ideology, media consumption, and age. Our findings suggest that public health messaging likely faces additional challenges from growing ideological cleavages in American politics.

## Theory

Different lines of research lead to four hypotheses for the influence of information by expert sources in the context of COVID-19. First, research shows the public relies on source cues to process information (Botero et al. 2015; Hartman and Weber 2009), assessing source credibility (Darmofal 2005) and often overlooking information credibility. That is, people are more persuaded by arguments given by experts – those possessing formal qualifications or experience on the issue (Thon and Jucks 2017). This reliance on expert cues could be exacerbated during a health pandemic, when people may suspend their many biases – instead prioritizing accuracy goals in line with their own safety. Indeed, a recent poll found that Americans highly trust medical workers (Archer and Ron-Levey 2020). This research would predict that people observe public health recommendations from those qualified to give them during the pandemic: experts.

Conversely, though, motivated reasoning *also* shapes people’s assessment of source credibility (Druckman and Mcgrath 2019). Directional motivated reasoning responds to intentional sentiment (Bullock et al. 2015) or subconscious affect (Taber and Lodge 2006), as cognitive processes are selected to reaffirm an existing world view. Previous literature focuses largely on politically motivated reasoning (Jerit and Barabas 2012; Flynn et al. 2017; Taber and Lodge 2006). This research would thus conversely predict that the downplaying of COVID-19 from the leader of the Republican Party (President Trump) would create a partisan divide – making nonpolitical source cues (and frames) meaningless for the transmission of information about COVID-19, where individuals would instead rely on political cues.

Third, today’s growing anti-intellectual sentiment – which creates a perceived conflict between experts and “common sense” (Merkley 2020) – may lead people to downplay health recommendations. We have indeed seen concerted efforts to delegitimize health officials and call into question their competence and trustworthiness during COVID-19, suggesting the pandemic is a context where anti-expert sentiment could guide both attitudes and behavior (Bursztyn et al. 2020). For example, people exposed to conservative media have been more likely to mistrust the Centers for Disease Control and Prevention (CDC), while trusting unfounded cures to combat COVID-19 (Jamieson and Albarracin 2020). This research would predict that information from experts could backfire.

Fourth, expert recommendations may also prime partisan identity. In recent years, Republicans have become skeptical of the scientific community (Hamilton, Harter and Saito 2015), as Republican elites associate institutions of higher education with partisan out-groups, and in doing so question their moral integrity (Motta 2018). Thus, this research would also predict that information from experts would backfire, but that this would be moderated by partisanship.

This paper uses a survey experiment to test these four source cue hypotheses – examining if, in the context of COVID-19: (1) people find experts persuasive; (2) people do *not* find experts persuasive; (3) people react negatively to experts; and/or (4) expert animus is particular among Republicans. Beyond source cues, though, we test if message framing matters in the context of COVID-19 – that is, if fact- or experience-based messages as well as gain- or loss-based messages either have a main effect or moderate the main effect. This additional variation is motivated by research that frames matter (Mondak 1993) – particularly that (1) narrative or testimonial information can be more persuasive than factual or evidence-based information (Braverman 2008) and (2) gain-framed health messages are considered more credible than loss-framed ones (Rothman et al. 2006).

## Survey experiment

In our survey design, each respondent received four vignettes in random order: (1) within-home social distancing, (2) prisoner release programs, (3) restarting college football, and (4) mail-in ballots. On each topic, we designed three sets of manipulations: expert versus nonexpert, fact versus experience, and gain versus loss.

For the first set of manipulations (expert vs. nonexpert), we embedded source cues by manipulating the person who delivered the statements: in the expert treatments, messages were provided by a physician, a former prison doctor, a public health expert, and an election official, respectively; whereas in the nonexpert treatments, messages were delivered by a recovering COVID-19 patient, a retired prison guard, an avid sports fan, and a voter, respectively.

For the second set of manipulations (fact vs. experience), we varied how people talked about the subject at hand: in the fact treatments, the individual stated “evidence shows that the most common means of passing on the virus is…,” whereas in the experience treatments, the individual stated “among the people I know that at one point had COVID-19…”

For the third set of manipulations (gain vs. loss), we varied whether complying with the rules would result in a gain or loss. For example, on the topic of social distancing, the messages were “from this [self-quarantine], they would benefit the health and safety of those close to them…” (gain) versus “failing to do so [self-quarantine] risks spreading the virus among those close to them” (loss). See full text in Appendix A.

Our expectation was that because of declining trust in experts, people would be more likely to be persuaded by nonexpert than expert sources.^1^ We also expected this effect to be moderated by issue frame, where experience- and gain-framed messages from nonexpert sources would be most persuasive.

Participants were recruited from the Lucid survey platform on May 20, 2020. On this day, 4,722 participants were recruited, although 36 were eliminated for various reasons (see Appendix E), leading to 4,686 participants (participant demographics by group in Appendix B). Participants were randomly assigned to one of the nine experimental conditions (eight treatments and a control group). After receiving pretreatment questions, participants read four vignettes from the same condition. Again, the conditions varied whether the source was an expert or nonexpert as well as whether the information was fact- or experience-based and framed as a gain or loss (see Table 1), and the four vignettes discussed within-home social distancing, prisoner release programs, restarting college football, and mail-in ballots.

**Table 1:** Assignment of Treatment Groups

Source Cue	Information			
	Fact		Experience	
	Gain	Loss	Gain	Loss
Expert	Group 1 (*N* = 498)	Group 2 (*N* = 503)	Group 3 (*N* = 495)	Group 4 (*N* = 536)
Nonexpert	Group 5 (*N* = 518)	Group 6 (*N* = 507)	Group 7 (*N* = 540)	Group 8 (*N* = 559)
Control group 9 (*N* = 554)				

The dependent variable was the incorporation of the information received, measured after each vignette by asking participants their views on the issue. Participants gave their responses on a seven-category Likert scale from strongly disagree to strongly agree (“How much do you agree with the following statement?”) in response to four statements: (1) “People should isolate in a separate room and stay away from family and other residents within the household if a family member suspects they were exposed to coronavirus or develops symptoms” (within-home social distancing); (2) “All states should expand prisoner release programs to release nonviolent offenders from prisons and other detention centers during the COVID-19 crisis” (prisoner release programs); (3) “The NCAA should decide to postpone this season’s kickoff for college football due to the COVID-19 crisis” (restarting college football); and (4) “All states and local governments should continue to have in-person rather than mail-in ballots for elections” (mail-in ballots).

To ensure attention to the vignettes, participants had to spend at least 15 seconds on each vignette before they were allowed to proceed and were also given a manipulation check (see Appendix A). After this, participants were asked about their levels of trust in various institutions and their personal experience with COVID-19. They were then debriefed and paid.

We note that in vignettes 2 and 3 (prisoner release programs and restarting college football), an error was made and “Dr.” remained in the name of both expert and nonexpert sources (although the descriptions demonstrated expertise and nonexpertise). We acknowledge that people may have considered the nonexpert as an expert because of this, thus biasing the results for these two vignettes (but leaving the other two vignettes valid). We thus remove these two vignettes from the analysis of expert versus nonexpert source cues.

## Results

**Figure 1:**
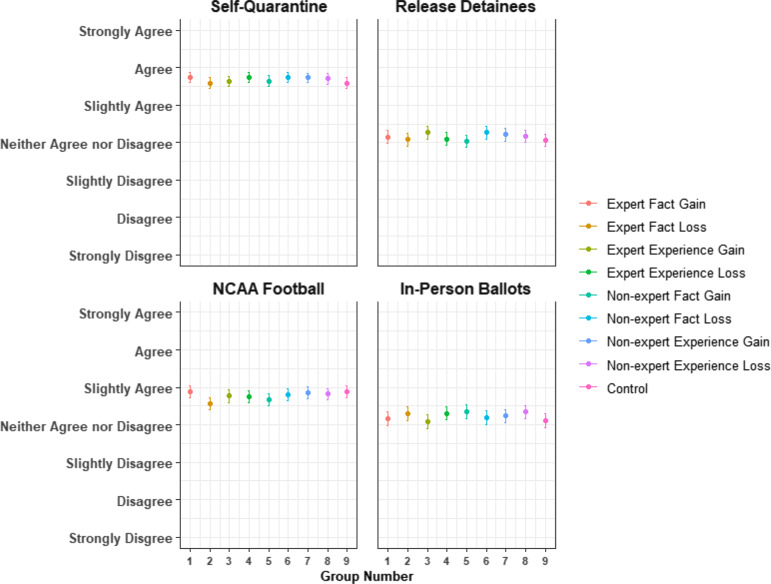
COVID-19 vignette response distributions by treatment condition. Notes: Subjects were asked how much they agree with a policy statement corresponding to each vignette. Response options ranged from strongly disagree to strongly agree. Points represent the mean response and intervals represent the 95% confidence intervals.

**Figure 2:**
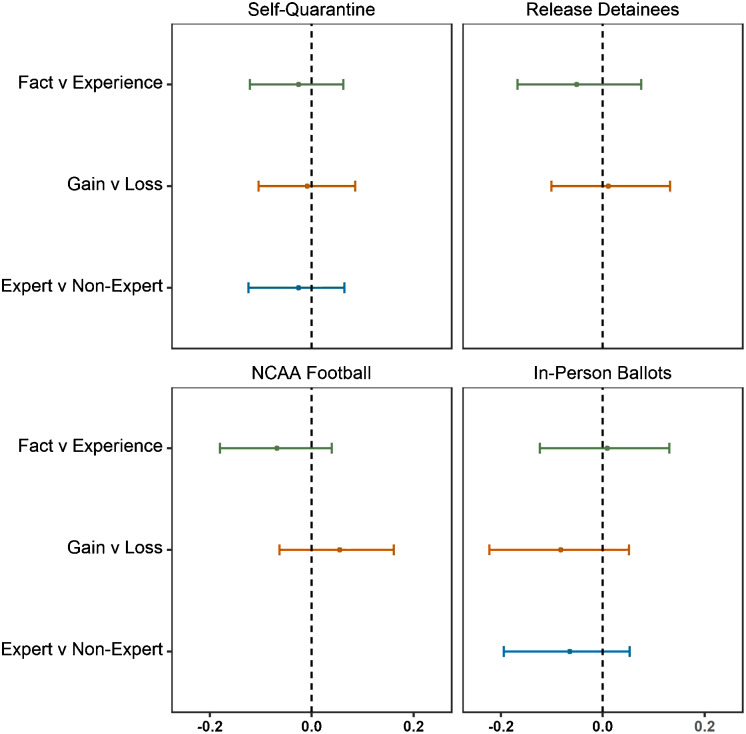
Difference in mean responses between test conditions. Notes: This figure provides the difference in mean policy agreement between the two conditions indicated in the y-axis label. Each condition is associated with the four treatment groups labeled in Table 1. 95% confidence intervals are derived from nonparametric bootstrapping. Vignettes on releasing detainees and NCAA football are excluded from the expert versus nonexpert analysis due to mistakenly adding a “Dr.” prefix for the nonexpert in the text of these vignettes.

As shown in Figures 1 and 2, the differences in mean responses between treatment groups are minimal. We find no significant effect of any of the eight treatments as compared to the control group for any of the four vignettes (see Appendix C: t-tests). Even when accounting for potential moderators (including partisanship), we find that neither source cue nor frame influenced people’s responses to questions about COVID-19. These null findings remain robust to nonparametric bootstrapping, lowering the manipulation check standards, accounting for low attention, and pooling treatments to increase statistical power (see Appendix C for robustness checks).

Note that: (1) our multiple comparisons increase the chance of a Type I error – detecting an effect where there is none – although we find zero effects; and (2) our experiment has the power (0.9) to detect a small effect size (Cohen’s *d* = 0.2) in each group, so the possibility of a Type II error – failing to detect a treatment effect where there is one – is quite low. An equivalence test using the “two-one-sided t-tests” (TOST) procedure yields significant results at the 0.95 levels for all inter-group and inter-treatment comparisons (Δ_*L*_ = –0.25, Δ^*U*^ = 0.25), suggesting that the true difference of means is zero (Lakens, Scheel, and Isager 2018). Thus, we cannot reject the null hypothesis that source cues and issue frames do not influence people’s responses to questions about COVID-19.

Bayesian factor analysis, comparing the relationships of expert to nonexpert source cues, fact to experience framing, gain to loss framing, and all treatments to the control group – each with the null of the respective relationships – also generally supports the null hypotheses. At the default prior distribution, a Cauchy distribution with the parameter γ set to 1, BF_01_, contrasting all treatments with the control, is 22.5 for the self-quarantine vignette and between 7.7 and 12.1 for the remaining three vignettes. The Bayesian factors model the change in posterior confidence from prior beliefs that the null hypothesis is more likely than the alternative hypothesis given the data and prior beliefs (see Appendix C: Bayesian factor analysis for factors graphed over a range of prior beliefs).

**Figure 3:**
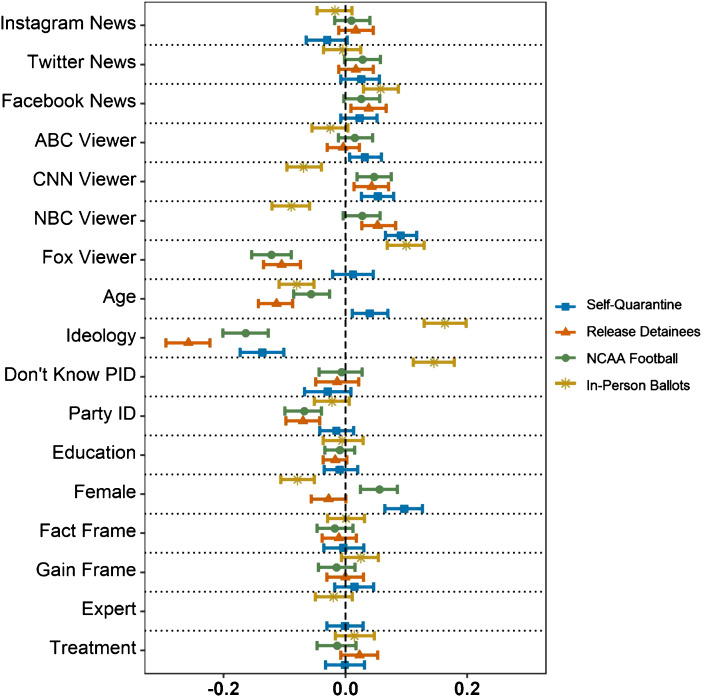
Responses to COVID-19 vignettes by observed variables. Notes: This figure displays OLS regression standardized coefficient estimates with respect to the dependent variable (agreement with the respective policy statement). Confidence intervals are derived from nonparametric bootstrapping with 95% confidence. Appendix C, Table 6 displays the corresponding regression table and variable descriptions.

Support of null relationships through Bayesian factors are even stronger regarding contrasts between treatment conditions. Across the four vignettes, BF_01_ associated with the expert to nonexpert contrast ranges from 25.1 to 39. With respect to the fact to experience contrast, BF_01_ ranges from 21.7 to 37.3. Lastly, with respect to the gain to loss contrast, BF_01_ ranges from 18 to 39.1. Suggestions for appropriate language to describe the strength of evidence indicate a “positive” to “substantial” level of support for the null when comparing treatments to control and a “strong” to “very strong” level of support for the null when comparing relevant treatment conditions to each other (Jarosz and Wiley 2014). See Appendix C: Bayesian factor analysis for regression model comparisons between the Figure 3 model above and the nested models removing each independent variable.

Lastly, while none of the manipulated variables meaningfully influenced participant responses, some of the observed variables – ideology, media consumption, and age – explain much of the variation in participants’ responses (see Figure 3).

## Discussion

We thus find no evidence that expertise of source cues or issue frames influence people’s responses to questions about COVID-19. However, we believe these null results do not negate the ample research on source cues and issue frames. In what follows, we address some design decisions that could have driven the null results as well as what we believe to be the more likely culprits: issue salience and political polarization.

First, it is possible that a more extreme difference between the expert and nonexpert source cues, experience versus fact, and/or gain versus loss would have led to treatment effects. The robust nulls that we find, however, suggest that even *if* a stronger treatment had *some* effect, the effect would be quite minimal.

Second, it is also possible that social desirability weakened potential treatment effects – the dire circumstances of a pandemic may have led to convergence on socially desirable norms, thus limiting variation in our dependent variable and undermining our ability to detect other patterns (Daoust et al. 2020). We did anticipate this, however, and thus collected preliminary data with a separate sample asking questions about COVID-19. We then reworded questions with low variance to limit social desirability effects (e.g., our questions ask about *others’* behavior rather than one’s own).

Further, the high variation in our ultimate survey responses suggest social desirability did not drive the null findings. Except for vignette 1, the distribution of the responses for all other vignettes has a negative excess kurtosis (<-1) and low skewness (between −0.5 and 0.5), suggesting that their distribution is symmetric and more fat-tailed than the normal distribution. Vignette 1 has a high peak (with most participants either strongly agreeing or agreeing with self-quarantine) but still has a relatively high standard deviation (*μ* = 2.32, *σ* = 1.57). It is still possible, of course, that social desirability muted potential effects, but, again, our robust null findings make us doubtful that social desirability is the whole story here.

Instead, the data – which show the strong influence of ideology and media consumption on views toward COVID-19 – suggest to us that the issue salience of, and political dynamic surrounding, the pandemic drove our null findings. Indeed, research suggests that salient issues are more ridden with directional goals (Jerit and Barabas 2012; Slothuus and de Vreese 2010), and although we attempted to choose less salient issues *within* the crisis, COVID-19 *itself* – a global health pandemic – was quite salient in May 2020.

Further, elites and media outlets were quick to adopt positions regarding COVID-19, creating a political dimension to the crisis that may have encouraged the public to fall on political lines. Trump’s quick downplaying of the dangers of COVID-19 – whether out of a motivation to avert panic or something less noble – undoubtedly held extraordinary weight for his supporters, who (like the rest of the public) had little information about the novel virus. Fox News indeed reinforced this idea that fears of the virus were overblown, something rarely seen in the liberal news media.

The COVID-19 pandemic (by nature) has created fear and uncertainty, so it is unsurprising if it has encouraged people to listen to the sources they trust – for some, those sources may be health experts, but for others those may be their preferred media outlet and/or political leaders. This choice of which sources to trust has likely been influential in how the pandemic has played out and how political it has become: indeed, we see that the viewing of two politically opposed media outlets (Fox News and NBC News) largely guided views toward COVID-19. This fits with research suggesting that when elites disagree with expert opinion, public opinion on the disputed issue will be divided by politics (Darmofal 2005).

Thus, it is possible that had our study been run in a less polarized environment (either a different country or earlier on in the crisis), source cues and issue frames could have mattered. The US remains a relative anomaly in the developed world, with political leaders politicizing health care recommendations and actively spreading misinformation. Deploying our study in environments with less affective polarization and misinformation, such as in Northwestern European countries (Reiljan 2020), or those with declining levels of affective polarization, like Germany or Norway (Boxwell et al. 2020), could have uncovered treatment effects.

Similarly, perhaps running our study earlier on in the crisis would have led to different findings. Keep in mind, however, that our study was run merely 2 months after the first US COVID-19 case announcement, suggesting attitudes crystallized quite rapidly.^2^ The fact that that the American public formed polarized attitudes so quickly in this health crisis highlights the strength of the ideological cleavages in the US as well as the frightening possibility that future advice by public health experts may be ineffective – even during a crisis that threatens lives, the economy, and our way of life. It suggests that not even a pandemic can take politics off the table.

An inability to communicate useful information to the public is always concerning – but in this case it has particularly negative outcomes, leading to higher rates of COVID-19 infection, hospitalization, and mortality. Our findings thus leave us doubtful that health communication will be effective at overcoming previous media and elite cues, at least in the US. Politicizing this health crisis may be unable to be undone – at least in the current information environment.

## Footnotes

1 This prediction is detailed in the original grant proposal and the preregistered report can be found in Appendix D.

2 Crystallized attitudes toward COVID-19 would help to explain our null findings, as it is possible that participants were already pretreated with cues and frames in the real world, diminishing the effect of cues and frames within the experiment (see Druckman and Leeper 2012).

## Supplementary material

For supplementary material accompanying this paper visit https://doi.org/10.1017/XPS.2021.3. click here to view supplementary material
