# Supplementary material for: The Effects of Source Cues and Issue Frames During COVID-19
Source: Journal of Experimental Political Science. 2021 Jan 29:1–10. doi: 10.1017/XPS.2021.3 (PMC7925984; doi:10.1017/XPS.2021.3)
Supplement: Supplementary file 1 [file S2052263021000038sup001.docx]

**APPENDIX A: Survey Experiment**

1. What gender do you most identify with? [Male / Female / Other (please specify)]
2. In what year were you born? [ ]
3. Please enter the zipcode of your place of residence: [ ]
4. What is the highest level of school you have completed or the highest degree you have received? [No high school / High School Graduate or G.E.D. / Some College but no degree / Associate Degree / Bachelor’s Degree / Postgraduate Degree / Professional Degree]
5. Thinking back over the last year, what was your family’s annual income (in USD)? [ ]
6. What of the following race or ethnic groups do you most identify with? [White, non-Hispanic / Black, non-Hispanic / Native American / Hispanic / Asian, Native Hawaiian, or other Pacific Islander / Middle Eastern / Other (please specify)]
7. Where would you place yourself on the following party identification scale? [strong Democrat / weak Democrat / Independent but lean Democrat / Independent / Independent but lean Republican / weak Republican / strong Republican]
8. Where would you place yourself on the following political ideology scale? [very liberal / liberal / slightly liberal / moderate / slightly conservative / conservative / very conservative / don’t know]
9. Which media sources do you follow, if any (list all that you regularly watch, listen to, or read? [ ]
10. What is your current religion, if any? [ ]

Please read the following statement and answer the question that follows.

**[Vignette 1]**

All Groups: Individuals that have contracted COVID-19 spread the virus through airborne droplets and surface contamination. Some suggest people should isolate themselves from others within their homes. [Groups 1, 2, 3, 4: A physician explained] [Groups 5, 6, 7, 8: A recovering COVID-19 patient explained] [Groups 1, 2, 5, 6: “Evidence shows that the most common means of passing on the virus is among those within the same home. If someone has symptoms or even believes they may have contracted COVID-19, they should self-quarantine for 14 days, including isolating in a separate room and staying away from family and other residents.”] [Groups 3, 4, 7, 8: “Among the people I know that at one point had COVID-19, the ones that did not spread the virus to their family and neighbors were those that quarantined. As soon as they experienced symptoms or were exposed to the virus, they isolated in a separate room and stayed away from family and other residents.”] [Groups 1, 3, 5, 7: From this they would benefit the health and safety of those close to them.”] [Groups 2, 4, 6, 8: Failing to do so risks spreading the virus among those close to them.”]

How much do you agree with the following statement, “People should isolate in a separate room and stay away from family and other residents within the household if a family member suspects they were exposed to coronavirus or develops symptoms?” [1 (strongly agree) to 7 (strongly disagree)]

**[Vignette 2]**

All Groups: There has been concern that prisons and immigration detention centers are particularly conducive to the spread of COVID-19. As a result, some state and local governments have released some nonviolent detainees. [Groups 1, 2, 3, 4: Dr. Matthew Gardner, A former prison doctor explained] [Groups 5, 6, 7, 8: Dr. Matthew Gardner, A retired prison guard explained] [Groups 1, 2, 5, 6: “The physical and functional characteristics of detention facilities are conducive to the spread of COVID-19. Detained populations and staff are in regular close contact and this has led to serious outbreaks.] [Groups 3, 4, 7, 8: “The physical and functional characteristics of detention facilities are conductive to the spread of COVID-19. I saw this every flu season. It seemed like half of the detainees and staff would catch it at the same time.] [Groups 1, 3, 5, 7: Releasing some detainees such as nonviolent offenders would help protect detained populations, staff and surrounding communities from COVID-19.”] [Groups 2, 4, 6, 8: Failing to release nonviolent offenders places a serious risk of a COVID-19 outbreak on detained populations, staff and surrounding communities.”]

How much do you agree with the following statement, “All states should expand prisoner release programs to release non-violent offenders from prisons and other detention centers during the COVID-19 crisis.” [1 (strongly agree) to 7 (strongly disagree)]

**[Vignette 3]**

All Groups: The NCAA is grappling with the decision on whether the season’s kickoff should proceed as normal this fall. [Groups 1, 2, 3, 4: Dr. Martell Blatt, an avid fan believes it’s in the best interest of the athletes and the economy to stick to its normal plan.] [Groups 5, 6, 7, 8: Dr. Martell Blatt, a public health expert believes it’s in the best interest of the athletes and the economy to stick to its normal plan.] [Groups 1, 3, 5, 6: “In my decades of experience attending games] [Groups 3, 4, 7, 8: “Several studies have shown that”], college communities can do what’s necessary to keep fans and athletes safe, whether that means screening fans at the gates or social distancing to mitigate the spread of COVID-19]. [Groups 1, 3, 5, 7: Opening the season on time will give a much-needed boost to the economy and the millions of fans around the country] [Groups 2, 4, 6, 8: Not opening the season will deprive the economy and millions of fans of a much-needed boost.”]

How much do you agree with the following statement, "the NCAA should decide to postpone this seasons kickoff for college football due to the COVID-19 crisis"? [1 (strongly agree) to 7 (strongly disagree)]

**[Vignette 4]**

All Groups: Some states and local governments are having in-person elections while others are shifting to mail-in voting in response to the COVID-19 pandemic. [Groups 1, 2, 3, 4: An election official explained] [Groups 5, 6, 7, 8: A voter explained][Groups 1, 2, 5, 6: “Because we share voting machines and come in close contact with others, just one infected person can contaminate many others while voting. Masks and distancing at polling locations can help, but as the pandemic is still active,] [Groups 3, 4, 7, 8: “I have spoken with individuals that have voted or worked polls since the COVID-19 outbreak. Some communities experienced outbreaks attributed to spread during in-person elections. Masks and distancing at polling locations can help, but as the pandemic is still active,] [Groups 1, 3, 5, 7: only moving elections to mail-in ballots will protect communities from the spread of coronavirus while preserving our democracy.”] [Groups 2, 4, 6, 8: if the government does not move elections to mail-in ballots, it not only increases the risk of coronavirus spreading but also risks the legitimacy of elections.”]

How much do you agree with the following statement, "all states and local governments should continue to have in-person rather than mail-in ballots for elections"? [1 (strongly agree) to 7 (strongly disagree)]

1. Which of the following topics did the survey discuss (click all that apply)? [Prison Release Programs / Mail-in Election Ballots / Wearing Masks in Restaurants / Quarantining within Homes / Small-Business Loans / Restarting College Football / Vitamin C Tablets for COVID-19]
2. How much do you trust the following sources for accurate information about COVID-19? [1 (strongly trust) 🡪 7 (not trust at all)]
   1. Center for Disease Control (CDC)
   2. Medical Doctors
   3. U.S. President
   4. Governor of your State
   5. Mayor/Local Council
   6. World Health Organization (WHO)
   7. National News Media
   8. Local Newspapers
   9. COVID-19 Patients
   10. Church/Religious Group
3. Have any of your family or close friends been diagnosed with COVID-19? [Yes / No / Maybe]
4. Please leave any additional comments or questions, if any: [ ]

**APPENDIX B: Survey Participants**

4,722 participants completed the survey. Of these, 36 responses were removed from the analysis, given that they did not answer the vignette questions. We present the median value of *Age* and the mean value of *Education* and *Ideology* in each group. As for the categorical variables, we provide the percentages of each level of those variables. The demographic details of the participants are below:

***Table 1. Participants and Demographic Details***

| **Traits** | **Group 1** | **Group 2** | **Group 3** | **Group 4** | **Group 5** | **Group 6** | **Group 7** | **Group 8** | **Group**  **9** |
| --- | --- | --- | --- | --- | --- | --- | --- | --- | --- |
| **Age** | 46  (16.8) | 41  (17.1) | 45.5  (16.5) | 45  (17.0) | 44  (17.4) | 44  (17.2) | 44  (17.4) | 46  (17.5) | 44  (16.6) |
| **Gender** |  |  |  |  |  |  |  |  |  |
| Female | 52.9% | 51.1% | 48.4% | 49.8% | 54.8% | 54.9% | 55.2% | 49.6% | 50.2% |
| Male | 46.7% | 48.7% | 50.8% | 50.2% | 44.8% | 44.9% | 44% | 50.2% | 49.5% |
| Other | 0.4% | 0.2% | 0.8% | -- | 0.4% | 0.2% | 0.7% | 0.2% | 0.1% |
| **Education** | 3.74  (1.55) | 3.85  (1.49) | 3.65  (1.52) | 3.86  (1.55) | 3.71  (1.54) | 3.76  (1.55) | 3.74  (1.59) | 3.78  (1.57) | 3.86  (1.56) |
| **Political Party** | | | | | | | | | |
| Dem | 45.5% | 39.6% | 45.1% | 37.5% | 38.9% | 41.7% | 44.5% | 39% | 41.9% |
| Rep | 30.7% | 32.8% | 28.3% | 31.8% | 32.5% | 30.5% | 29.2% | 31.2% | 33.8% |
| Ind | 5.9% | 7.4% | 7.9% | 6% | 6.6% | 6.2% | 7.6% | 7.5% | 8.5% |
| Other | 18% | 20.3% | 18.6% | 24.7% | 22% | 21.6% | 18.6% | 22.3% | 15.8% |
| **Ethnicity** | |  |  |  |  |  |  |  |  |
| White | 72.3% | 69.1% | 65.9% | 70.5% | 71.7% | 72% | 67.5% | 71.6% | 69.1% |
| Black | 8.7% | 10.2% | 12.6% | 10.9% | 10.3% | 11.4% | 13.4% | 9% | 12.7% |
| Hispanic | 8.3% | 8.6% | 9.3% | 6.6% | 8% | 8.2% | 9.5% | 8.3% | 7.6 % |
| Asian or native Hawaiian | 5.7% | 6.2% | 4.9% | 5.4% | 5.3% | 4.4% | 4.1% | 3.8% | 4.9% |
| Middle Eastern | 0.6% | 0.4% | 0.4% | -- | -- | 0.8% | 0.2% | 0.9% | 0.2% |
| Native American | 1% | 0.8% | 1.2% | 1% | 0.4% | 0.2% | 0.7% | 1.4% | 1% |
| Other | 3.8% | 4.8% | 5.7% | 5.6% | 4.3% | 3% | 4.5% | 5% | 4.4% |
| **Ideology** | 4.05  (1.79) | 4.01  (1.82) | 4.04  (1.81) | 4.17  (1.87) | 4.21  (1.78) | 4  (1.85) | 3.94  (1.80) | 4.12  (1.85) | 4.03  (1.79) |
| **N = 4,686** |  |  |  |  |  |  |  |  |  |

**APPENDIX C: Robustness Tests**

**T-tests**

The dependent variable used for the subsequent analysis is agreement with the policy statement that responds to the respective vignette. Participant responses of “strongly disagree,” “disagree,” “somewhat disagree,” “neither agree nor disagree,” “somewhat agree,” “agree,” and “strongly agree” are coded as 1 to 7, respectively. The mean values of all groups are given in *Table 2*, below. We performed difference-in-means tests between all groups (separately) and the control group; between each group and its theoretical opposite (each expert group versus each non-expert group, each fact group versus each experience group, and each loss group versus each gain group); and between each pooled treatment set (all expert groups versus all non-expert groups, all fact groups versus all experience groups, and all loss groups versus all gain groups). None of the means were found to be statistically significantly different at the 0.05 levels. See means and standard deviations below:

***Table 2: Group Means (Standard Deviations in Parenthesis)***

| **Vignette** | **Group** | | | | | | | | | **Pooled** |
| --- | --- | --- | --- | --- | --- | --- | --- | --- | --- | --- |
|  | **1** | **2** | **3** | **4** | **5** | **6** | **7** | **8** | **9** |  |
| **1** | 5.74(1.5) | 5.59  (1.6) | 5.64  (1.6) | 5.74  (1.6) | 5.65  (1.6) | 5.74  (1.5) | 5.73  (1.5) | 5.7  (1.6) | 5.59  (1.7) | 5.68  (1.6) |
| **2** | 4.15  (2.0) | 4.09  (2.0) | 4.27  (2.0) | 4.10  (2.0) | 4.04  (2.0) | 4.27  (2.0) | 4.21  (1.9) | 4.17  (2.0) | 4.06  (2.0) | 4.15  (2.0) |
| **3** | 4.88  (1.8) | 4.56  (1.9) | 4.77  (1.9) | 4.75  (1.8) | 4.66  (1.9) | 4.8  (1.9) | 4.85  (1.8) | 4.82  (1.9) | 4.88  (1.9) | 4.78  (1.9) |
| **4** | 4.17  (2.1) | 4.3  (2.1) | 4.07  (2.1) | 4.31  (2.1) | 4.34  (2.1) | 4.19  (2.2) | 4.23  (2.1) | 4.34  (2.1) | 4.11  (2.2) | 4.23  (2.1) |
| **Pooled** | 4.73  (2.0) | 4.64  (2.0) | 4.69  (2.0) | 4.72  (2.0) | 4.67  (2.0) | 4.75  (2.0) | 4.76  (1.9) | 4.76  (2.0) | 4.66  (2.0) | 4.71  (2.0) |

**Non-Parametric Bootstrapping**

In order to ensure robustness of the tests, we also derived confidence intervals of the difference-in-means through non-parametric bootstrapping. The bootstrapping approach used for all difference-in-means and difference-in-difference calculations take 1,000 random draws of the survey respondents with replacement. The 95% confidence interval cut-offs are the 0.025 and 0.975 quantiles of the difference-in-means between treatment groups within the 1,000 draws or, for difference-in-difference confidence intervals, the difference between the difference-in-means among the relevant comparison groups.

As *Figure 1* (below) shows, none of the treatments were found to be significantly different from 0 at the 0.05 levels. Below we check for interactions between each pair of treatment conditions. The data is subset across each pair of individual treatment condition and difference-in-means treatment effects are calculated across each of the remaining two treatment comparisons within those subsets.

***Figure 1: Treatment Interaction Effects***


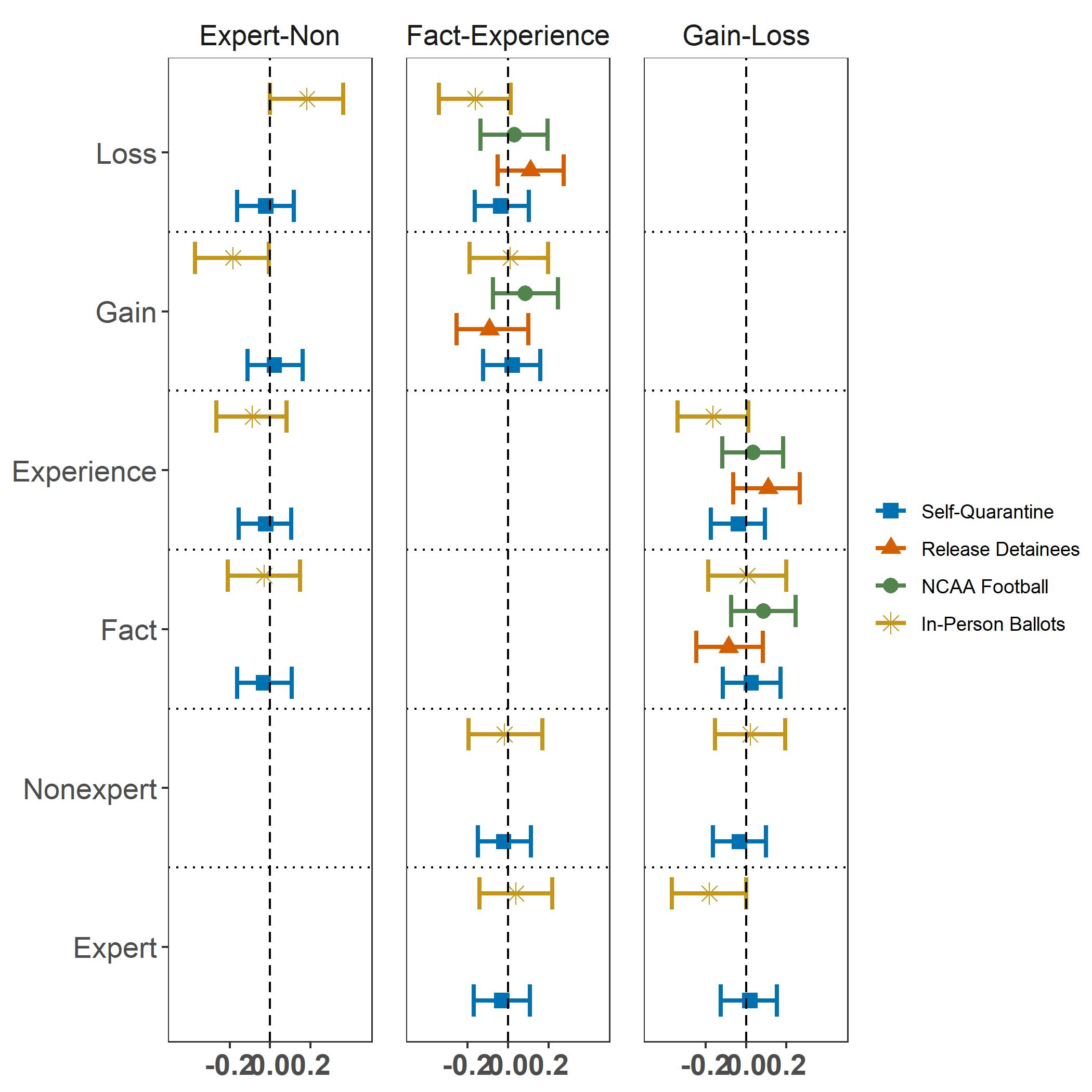


*The figure provides the difference in policy agreement mean responses between the treatment groups associated with the first condition indicated in the vertical column label and the treatment groups associated with the second respective condition. Groups selected for the vertical column comparison are subset according to the vertical axis. For example, the top left segment of the figure provides the difference in treatment effect between expert and non-expert source cues, but only among those that also received a loss frame. loss frame. The control group is not included among any categories. Confidence intervals are derived from nonparametric bootstrapping with 95% confidence.*

To see if partisanship is moderating the effect of the treatments, we check for whether interacting the treatments with partisanship changes our results. Only the difference in difference between fact and experience within the self-quarantine vignette displays a significant result. Overall, there is no discernible pattern. See *Figure 2*, below.

***Figure 2: Treatment Effects by Partisanship with Difference-in-Difference***


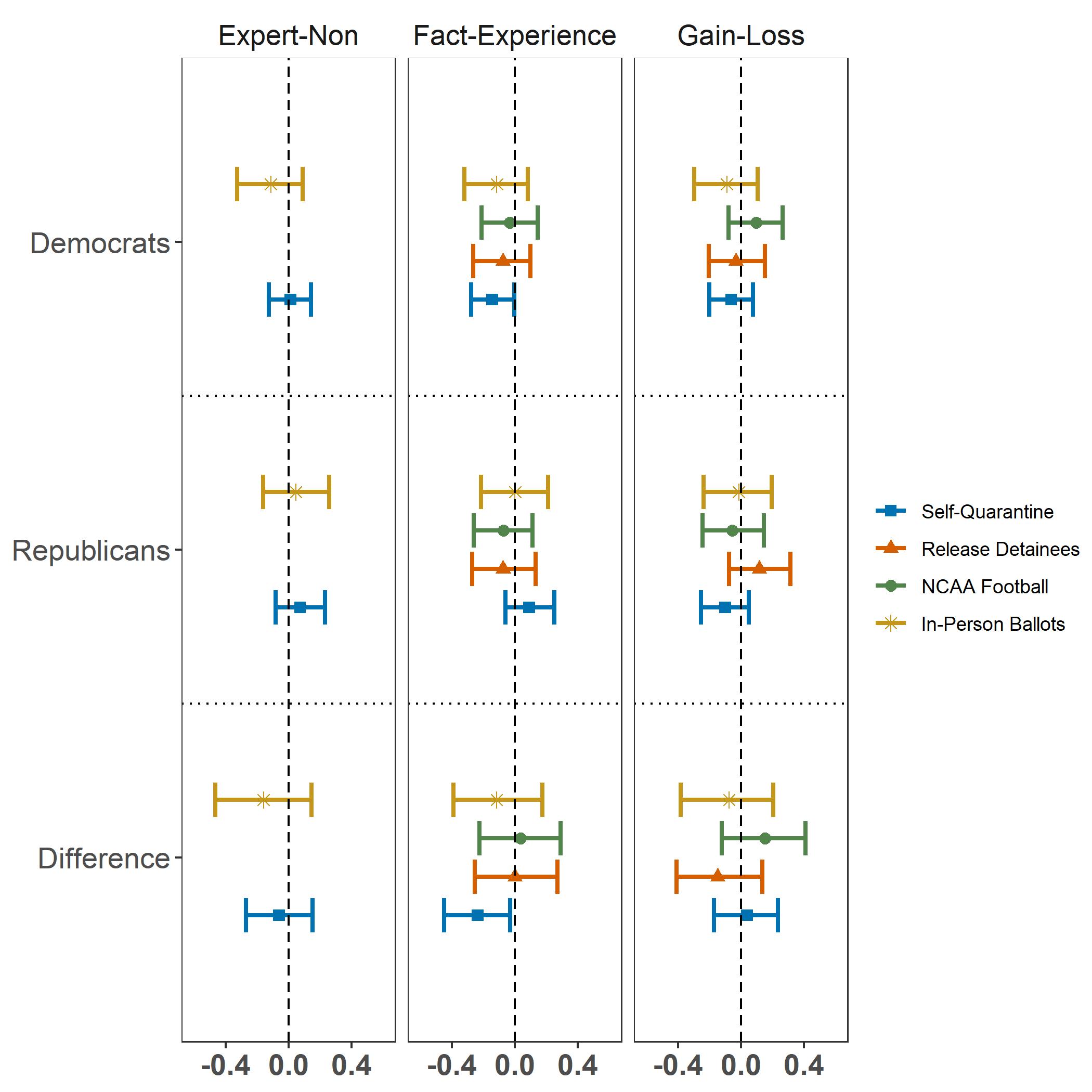


*The figure provides the difference in policy agreement mean responses broken down by party between the four treatment groups associated with the first condition indicated vertical column labels and the four treatment groups associated with the second respective condition. The bottom segment, labeled ‘Difference,’ provides the difference in difference of mean response between the two included party affiliations. The control group is not included among any categories. Confidence intervals derived from nonparametric bootstrapping with 95% confidence.*

**Manipulation Check**

In order to ensure sanctity of our responses, as a manipulation check, we asked participants to identify the 4 issues discussed in the survey out of a bank of 7 topics. Participants were then scored on the number of errors within this check, inclusive of both false negatives and false positives. Of the 4,722 respondents that completed the survey, 2,384 (50.5%) had zero errors, 3,340 (70.7%) scored zero or one error, 3,574 (75.7%) scored two or less errors, and 4,237 (89.7%) scored three or fewer errors. This jump is mostly attributable to those that only checked a single correct box among the seven choices. People who scored low in the manipulation checks were evenly divided across the experiment groups. *Figure 3*, below, provides the treatment effects removing those respondents that failed the manipulation check at various thresholds.

***Figure 3: Treatment Effects under Various Manipulation Check Thresholds***


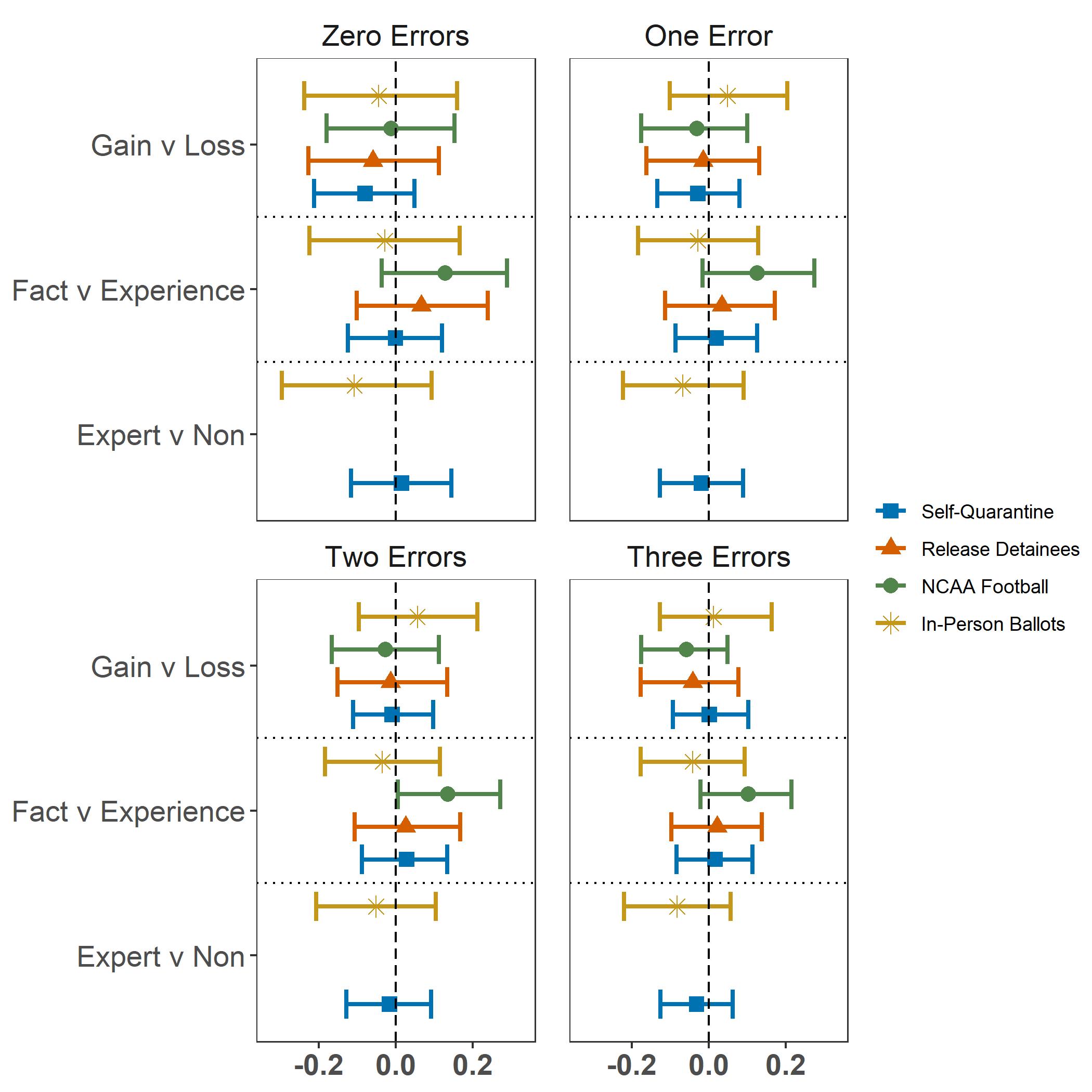


*The figure provides the difference in policy agreement mean responses between the four treatment groups associated with the first condition indicated in the y-axis label and the four treatment groups associated with the second respective condition. The control group is not included among any categories. Confidence intervals derived from nonparametric bootstrapping with 95% confidence. Vignette on releasing detainees and NCAA football are excluded from the expert vs. non-expert analysis, due to mistakenly adding a “Dr.” prefix for the non-expert in the text of these vignettes.*

We also identified participants who provided invalid or badly misspelled responses to open-ended questions, such as religion and zip code. These individuals, along with responses from a small number of duplicated IP addresses, were also coded as low attention. *Figure 4*, below, removes the respondents that we identified as low attention based on these open-ended responses along with incorporating various manipulation check thresholds. Of the 4,722 respondents that completed the survey 3,854 (81.6%) of respondents remain after removing those identified as low attention. Combining low attention with the various thresholds for the manipulation check leaves 2,164 (45.9%) of respondents at the zero errors threshold, 2,998 (63.5%) of respondents with one or fewer errors, 3,168 (67.1%) with two or fewer errors and 3,585 (7%) with three or fewer errors.

***Figure 4: Treatment Effects Removing Low Attention and Various Manipulation Check Thresholds***


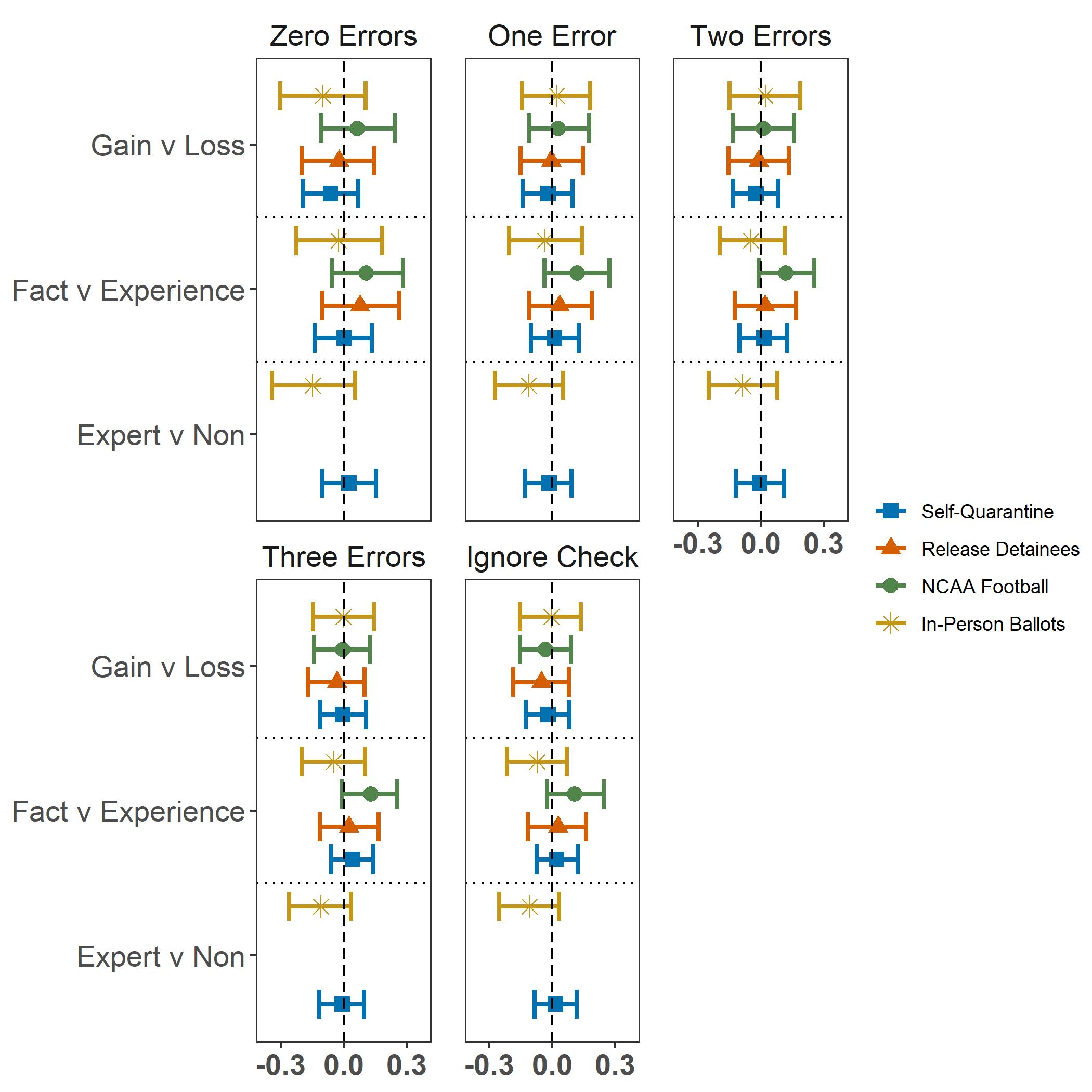


*The figure provides the difference in policy agreement mean responses between the four treatment groups associated with the first condition indicated in the y-axis label and the four treatment groups associated with the second respective condition. The control group is not included among any categories. Confidence intervals derived from nonparametric bootstrapping with 95% confidence. Vignette on releasing detainees and NCAA football are excluded from the expert vs. non-expert analysis, due to mistakenly adding a “Dr.” prefix for the non-expert in the text of these vignettes.*

In addition to the isolated treatment effects, *Figure 5*, below, provides the OLS coefficient estimates for treatments and other relevant variables using various low attention and manipulation check thresholds. The general findings are similar to those produced by all respondents, despite dropping a large proportion of cases. “Hi Focus” indicates remaining respondents after removing low attention and “Three Errors” indicates respondents that scored three or fewer errors on the manipulation check.

***Figure 5: Ordinary Least Squared Regression Coefficients with Various Manipulation and Low Attention Thresholds of Case Removal***


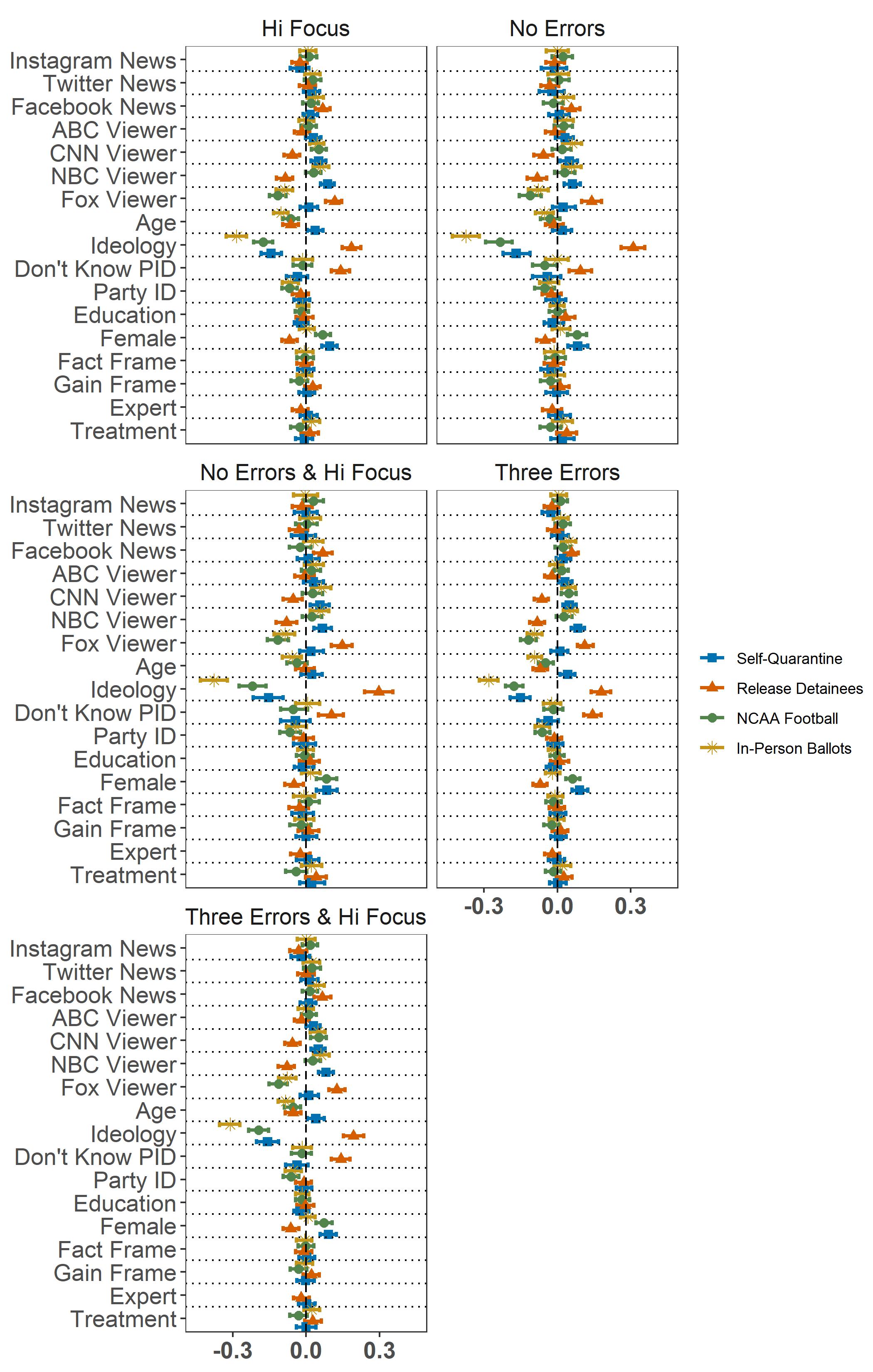


*The figure displays OLS regression standardized coefficient estimates with respect to the DV, agreement with the respective policy statement (see above footnote). confidence intervals are derived from nonparametric bootstrapping with 95% confidence. Table 6 below displays the corresponding OLS regression table accompanied with variable descriptions.*

***Figure 6: Treatment Effects Removing Quick Survey Responses at Relaxed Threshold***


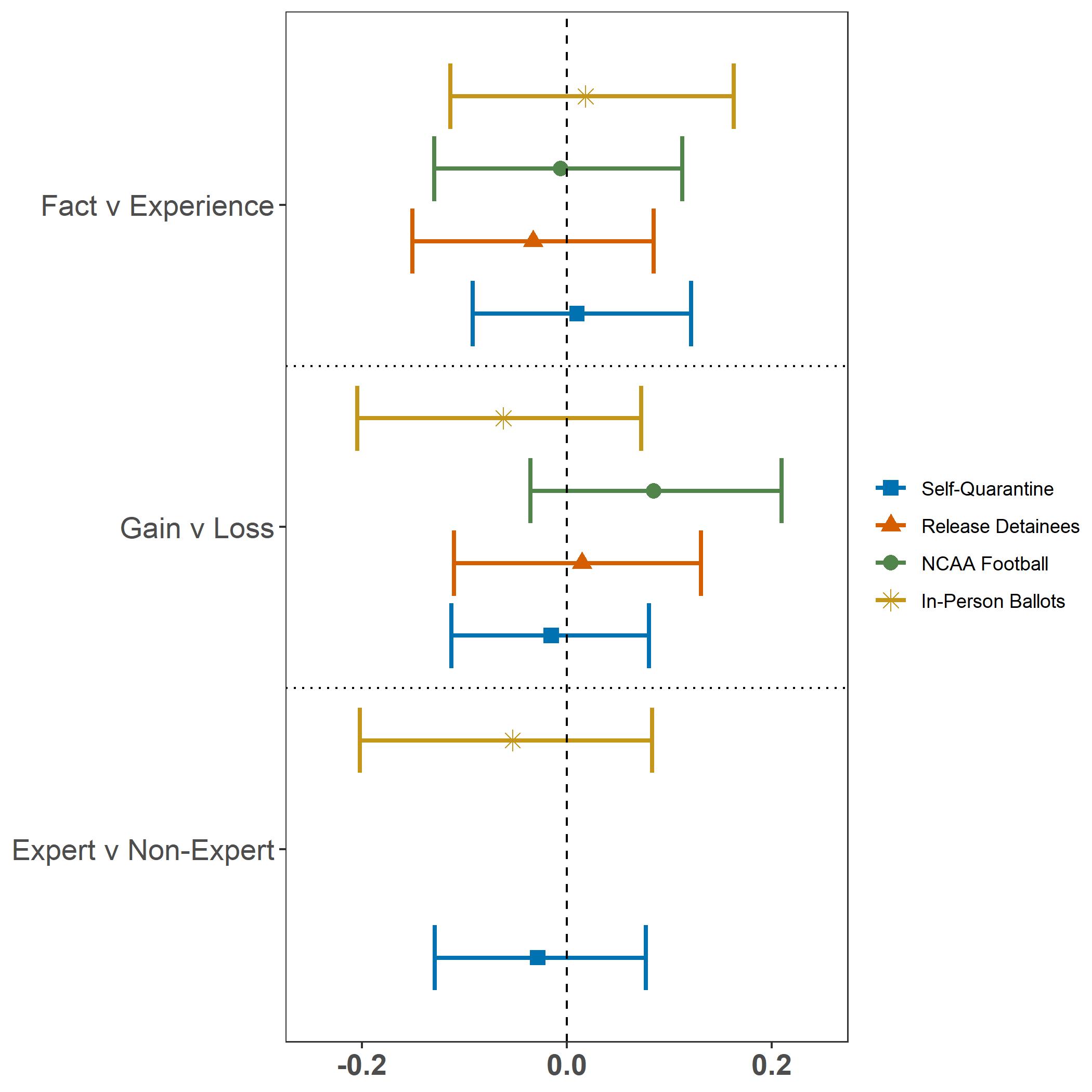


*The figure provides the difference in policy agreement mean responses between the four treatment groups associated with the first condition indicated in the y-axis label and the four treatment groups associated with the second respective condition. The control group is not included among any categories. Respondents that took less than 209 seconds to complete the survey are removed from the data. This threshold is chosen as an amount comparable to one standard deviation below the mean if duration was normally distributed, indicating that 15.8% of respondents are removed. Confidence intervals derived from nonparametric bootstrapping with 95% confidence. Vignette on releasing detainees and NCAA football are excluded from the expert vs. non-expert analysis, due to mistakenly adding a “Dr.” prefix for the non-expert in the text of these vignettes.*

***Figure 7: Treatment Effects Removing Quick Survey Responses at Strict Threshold***


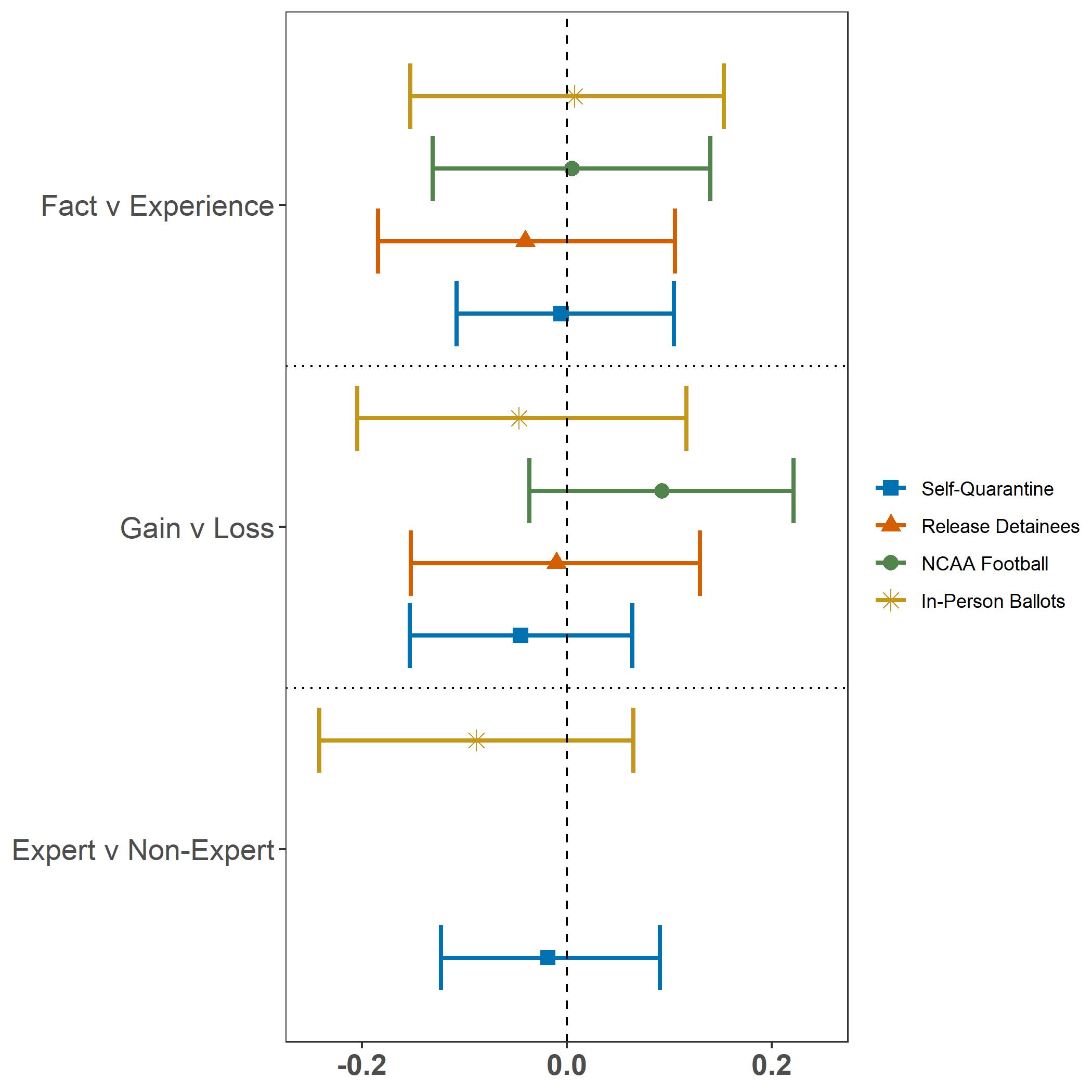


*The figure provides the difference in policy agreement mean responses between the four treatment groups associated with the first condition indicated in the y-axis label and the four treatment groups associated with the second respective condition. The control group is not included among any categories. Respondents that took less than 248 seconds to complete the survey are removed from the data. This threshold is chosen as an amount comparable to one standard deviation below the mean if duration was normally distributed, indicating that 30.8% of respondents are removed. Confidence intervals derived from nonparametric bootstrapping with 95% confidence. Vignette on releasing detainees and NCAA football are excluded from the expert vs. non-expert analysis, due to mistakenly adding a “Dr.” prefix for the non-expert in the text of these vignettes.*

**Bayesian Factor Analysis**

We calculate Bayesian Factors comparing difference in policy agreement between relevant treatment groups to null findings, the lack of a difference between those groups. Figures 8-11 show the results for the Self-Quarantine, Release Detainees, NCAA Football and In-Person Ballot vignettes respectively. The relevant contrasted treatment groups displayed in each of these figures are all expert conditions contrasted with non-expert conditions, fact conditions contrasted with experience conditions, gain conditions contrasted with loss conditions and all treatment conditions contrasted with the control group. The figures present BF_10_, the inverse of BF_01_ as reported in the main body of the paper for ease of interpretation. The data points at γ = 1 indicate a commonly used default prior for bivariate Bayesian Factor t-test analysis. A curve indicating the prior distribution ranging the γ variance parameter from .01 to 2 allows the reader to view changes in the Bayesian Factor that occur as prior probability changes. The figures indicate that unless prior belief already heavily leans towards the null hypothesis, posterior belief, given the collected survey experiment data, should shift expectations of the true relationship between the contrasted treatment conditions towards a higher probability of a null relationship.

***Figure 8: Self-Quarantine Vignette Bayes Factors Comparing Relevant Treatment Conditions Across Prior Distributions***


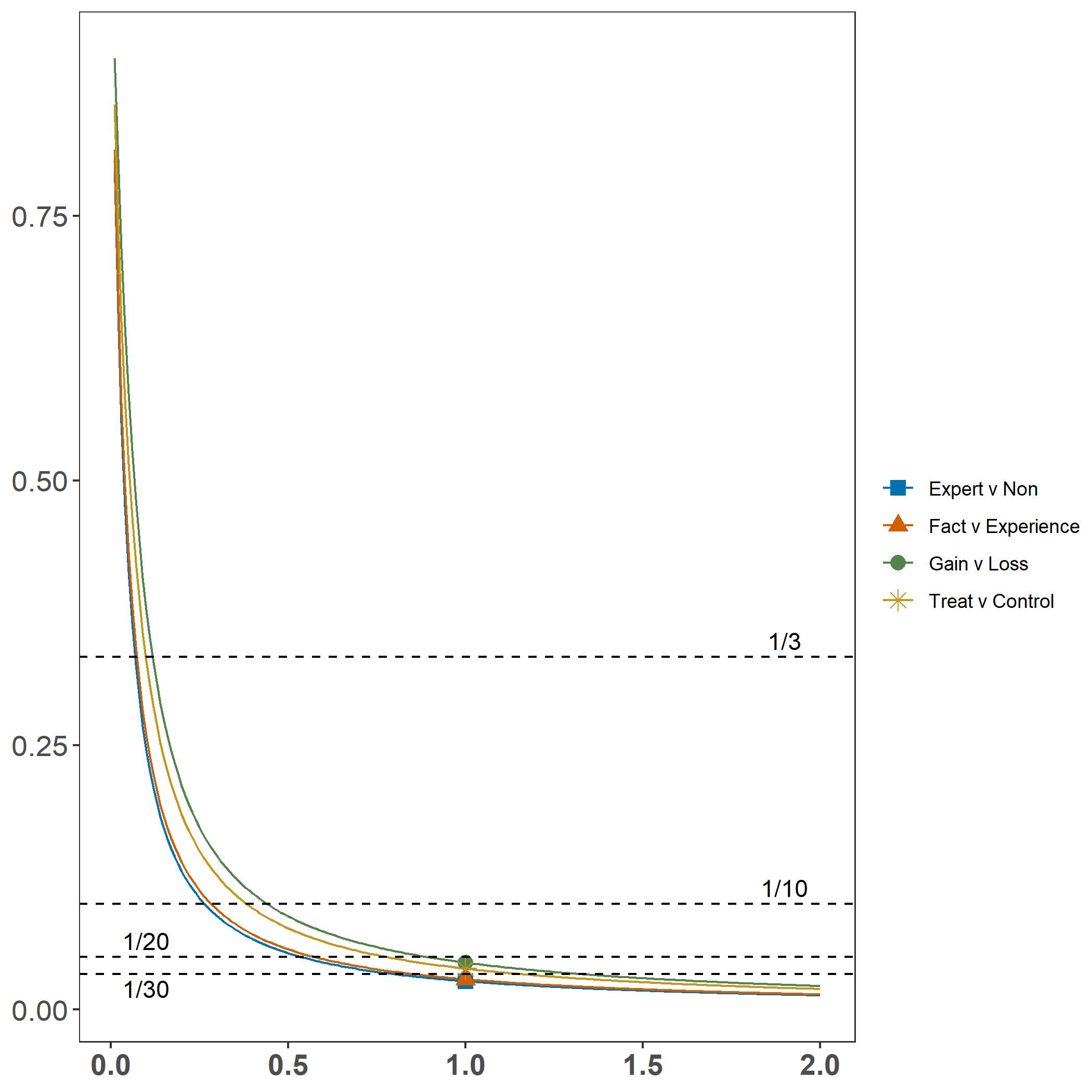


*The vertical axis represents the Bayesian Factor BF_10_. The horizontal axis indicates the γ parameter within Cauchy Distributions that serve as the prior probability. Dashed lines indicate common confidence thresholds applied to Bayes Factors.*

***Figure 9: Release Detainees Vignette Bayes Factors Comparing Relevant Treatment Conditions Across Prior Distributions***


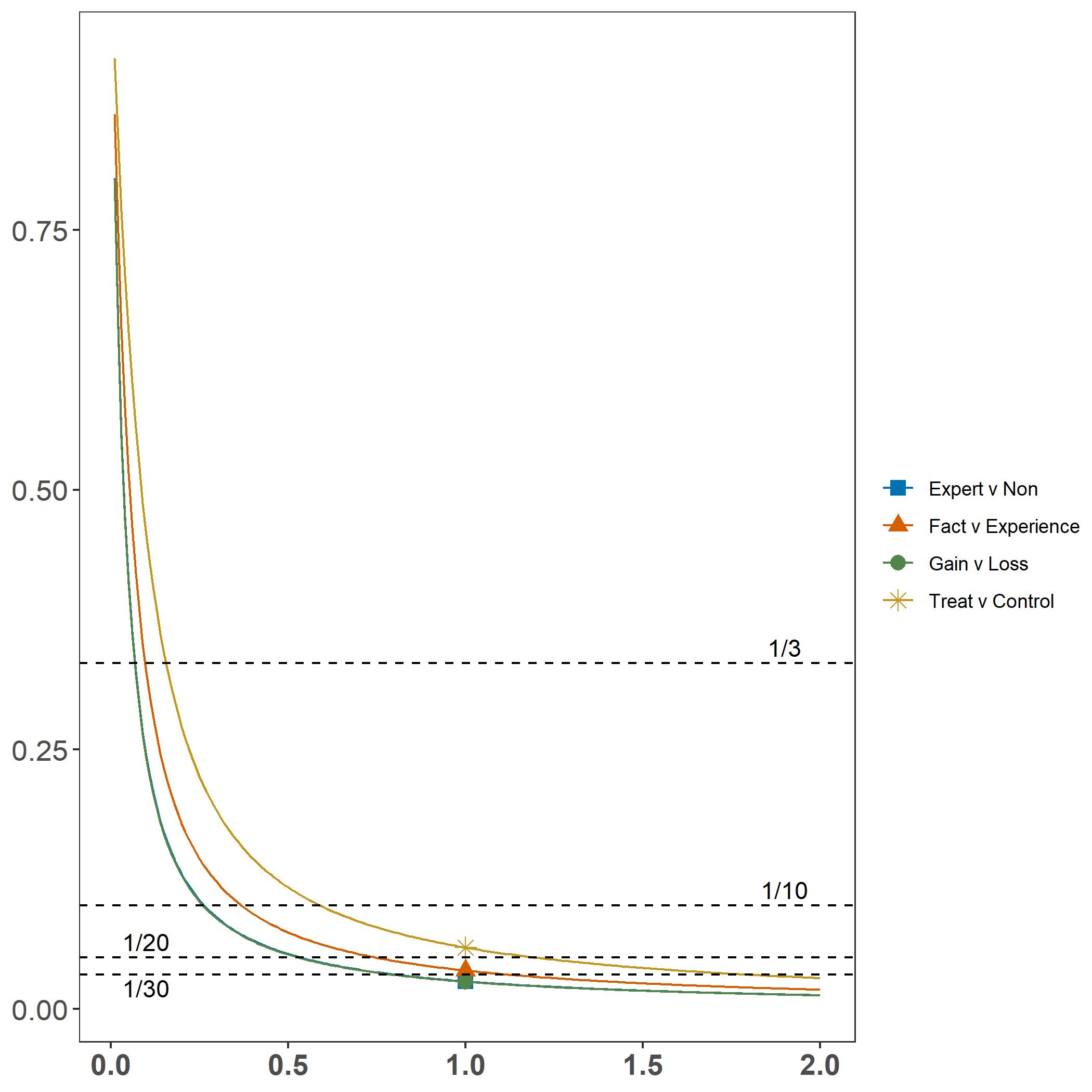


*The vertical axis represents the Bayesian Factor BF_10_. The horizontal axis indicates the γ variance parameter within Cauchy Distributions that serve as the prior probability. Dashed lines indicate common confidence thresholds applied to Bayes Factors.*

***Figure 10: NCAA Football Vignette Bayes Factors Comparing Relevant Treatment Conditions Across Prior Distributions***


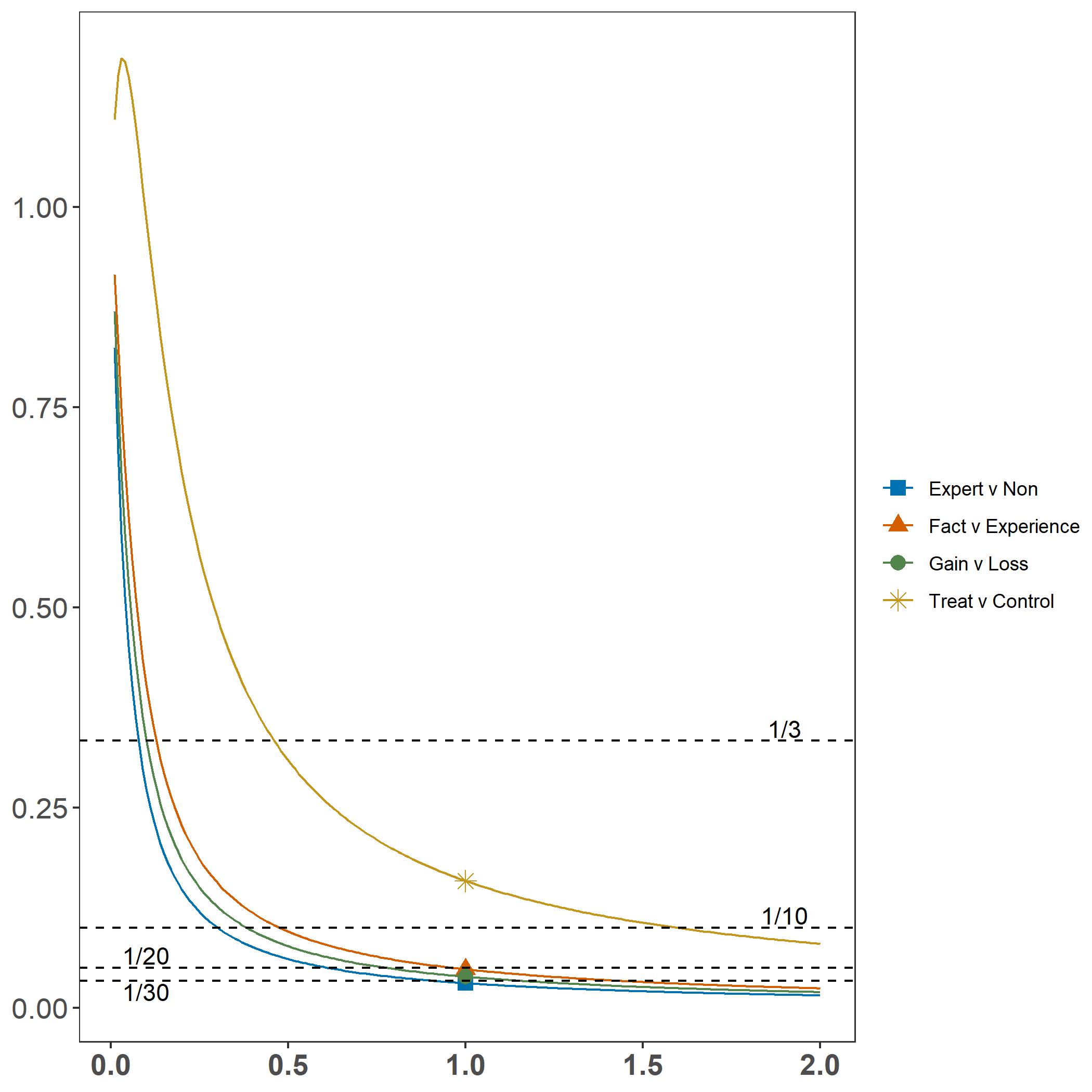


*The vertical axis represents the Bayesian Factor BF_10_. The horizontal axis indicates the γ parameter within Cauchy Distributions that serve as the prior probability. Dashed lines indicate common confidence thresholds applied to Bayes Factors.*

***Figure 11: In-Person Ballots Vignette Bayes Factors Comparing Relevant Treatment Conditions Across Prior Distributions***


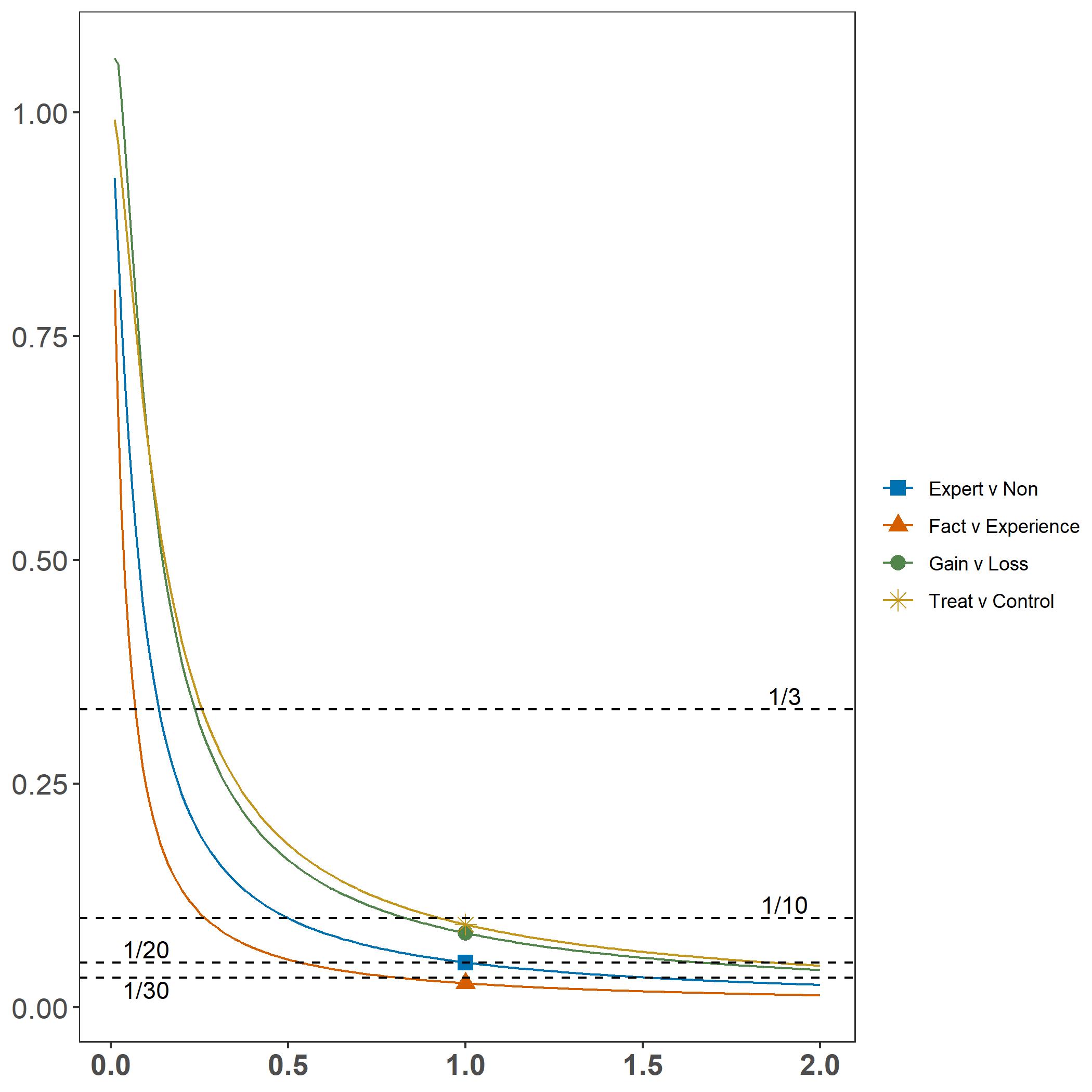


*The vertical axis represents the Bayesian Factor BF_10_. The horizontal axis indicates the γ parameter within Cauchy Distributions that serve as the prior probability. Dashed lines indicate common confidence thresholds applied to Bayes Factors.*

We also provide OLS regression nested model Bayesian Factor comparisons. Figures 12-15 display the proportional change in the Bayesian Factor when omitting individual variables, calculated by dividing the full model Bayesian Factor by the Bayesian Factor removing the respective variable from the OLS regression model. Changes below zero indicate that the additional explanatory power of the full model associated with the relevant variable is sufficiently large to overcome the Bayesian Factor preference for simplicity. In other words, the model is better fitted with the respective variable included. In contrast, changes above zero indicate that any additional explanatory power added by the respective variable in the full model is relatively small, being unable to overcome the built-in penalty for complexity in the Bayesian Factor calculation. Points indicate the Bayesian Factor comparisons at the default prior for regression model comparisons, while the intervals represent Bayesian Factors calculated at twice and one half of the default prior respectively. The nested model comparisons generally confirm the null finding, though at lower levels of certainty than the bivariate analysis above.

***Figure 12: Self-Quarantine Vignette Change in Regression Model Bayes Factor When Omitting Individual Variables***


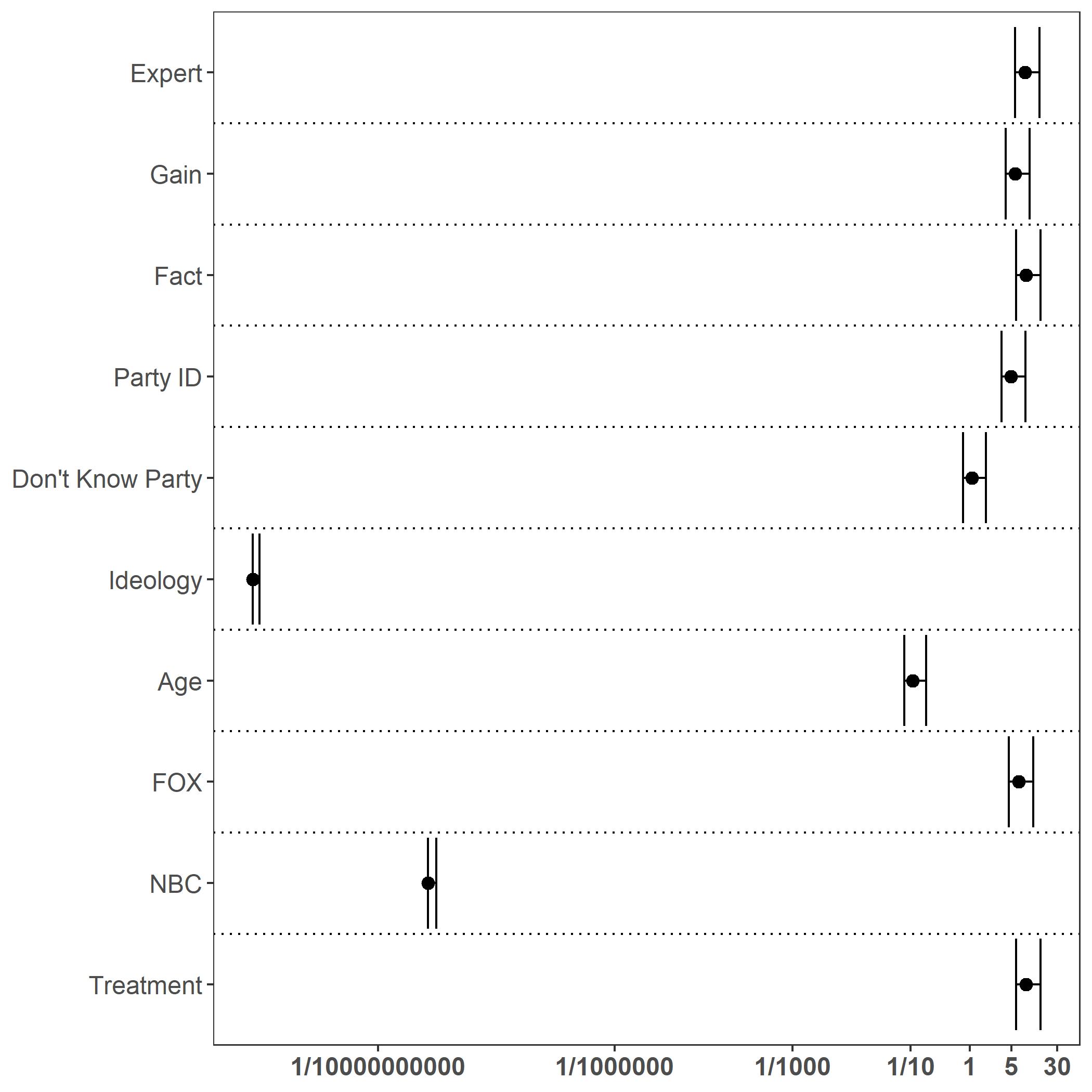


*The horizonal axis scale and Bayesian Factor values are plotted as natural logs for visual purposes. Tick marks on the horizontal axis are inverse logged to represent the original Bayesian Factor values. Vertical axis variable descriptions are available accompanying Table 6 below.*

***Figure 13: Release Detainees Vignette Change in Regression Model Bayes Factor When Omitting Individual Variables***


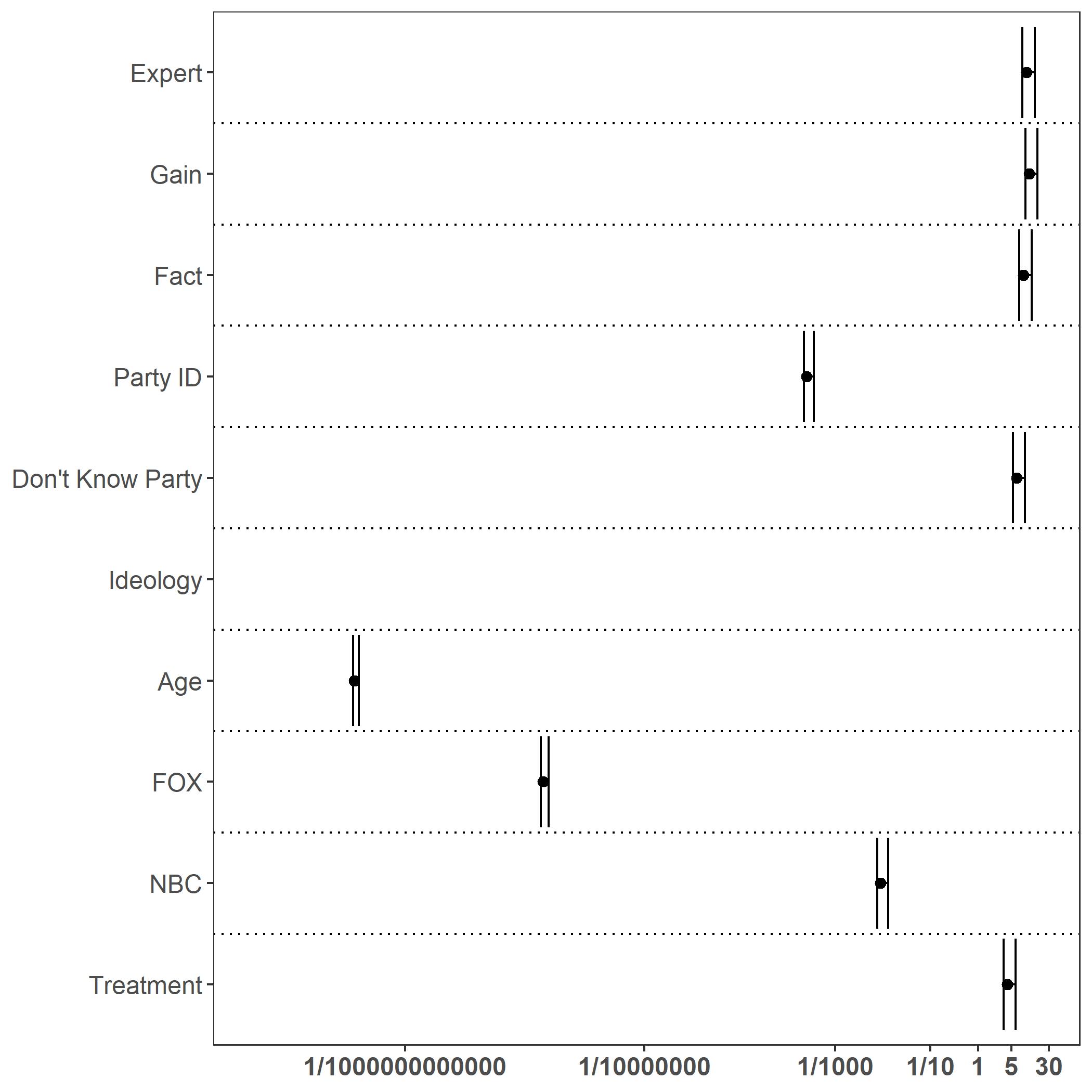


*The horizonal axis scale and Bayesian Factor values are plotted as natural logs for visual purposes. Tick marks on the horizontal axis are inverse logged to represent the original Bayesian Factor values. Vertical axis variable descriptions are available accompanying Table 6 below.*

***Figure 14: NCAA Football Vignette Change in Regression Model Bayes Factor When Omitting Individual Variables***


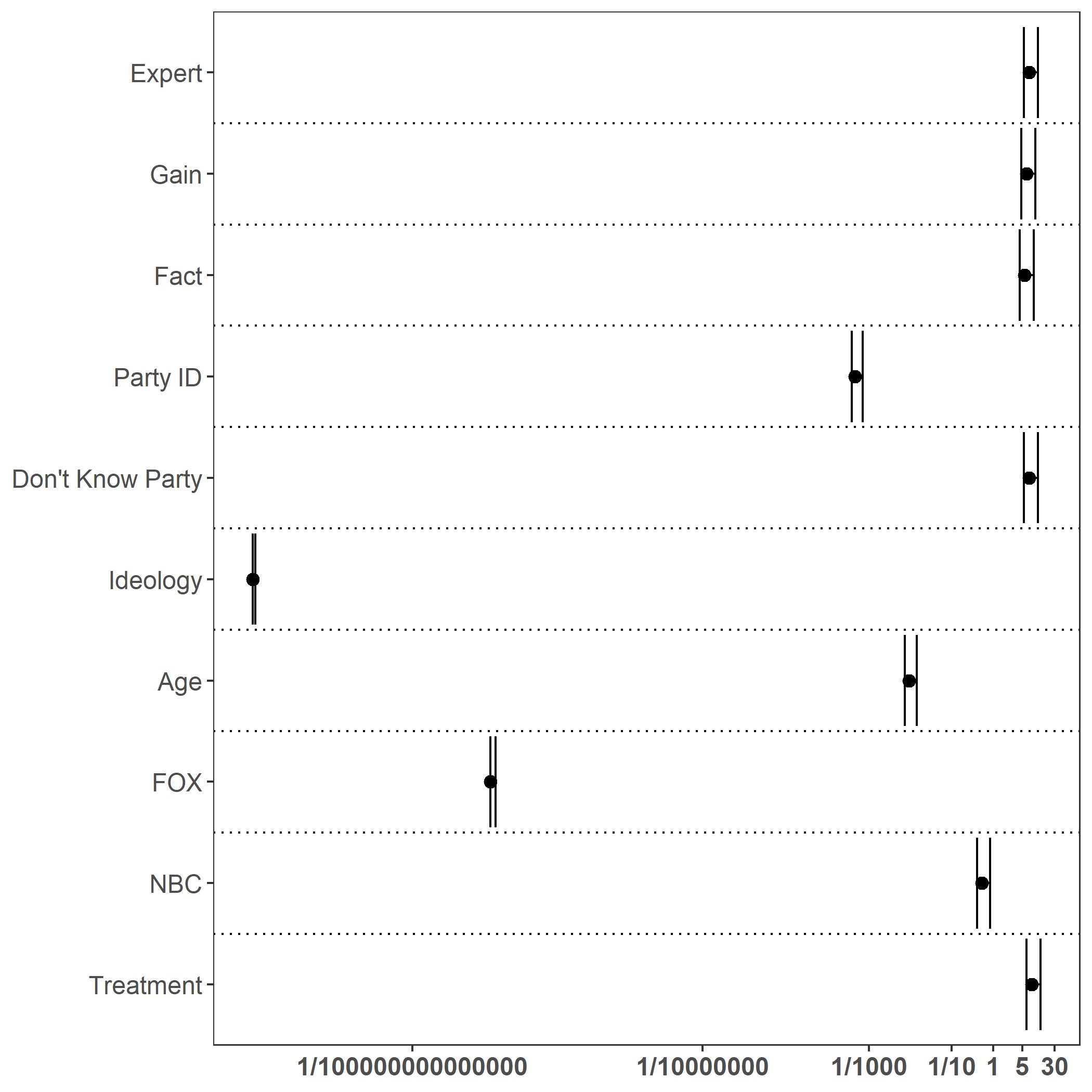


*The horizonal axis scale and Bayesian Factor values are plotted as natural logs for visual purposes. Tick marks on the horizontal axis are inverse logged to represent the original Bayesian Factor values. Vertical axis variable descriptions are available accompanying Table 6 below.*

***Figure 15: In-Person Ballots Vignette Change in Regression Model Bayes Factor When Omitting Individual Variables***


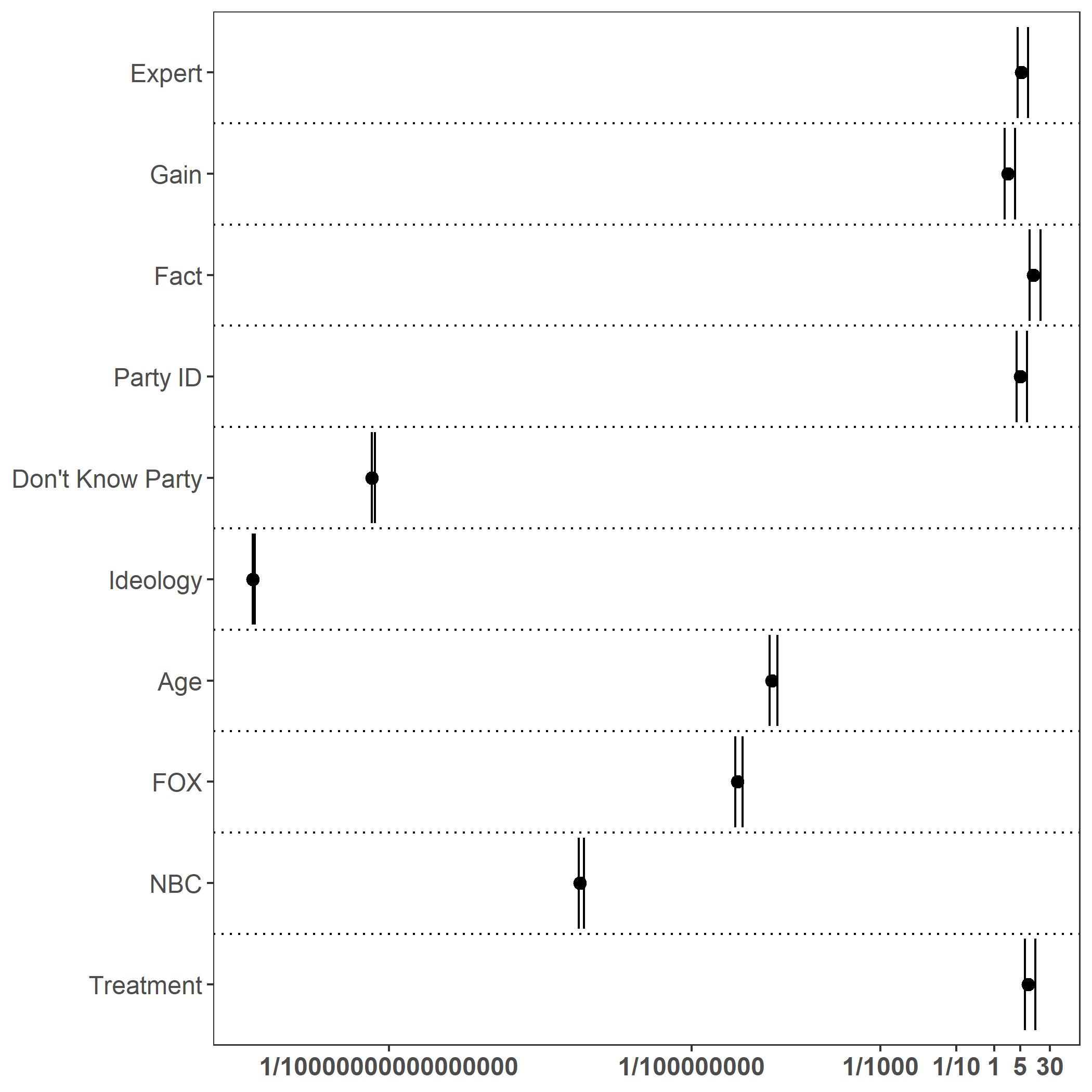


*The horizonal axis scale and Bayesian Factor values are plotted as natural logs for visual purposes. Tick marks on the horizontal axis are inverse logged to represent the original Bayesian Factor values. Vertical axis variable descriptions are available accompanying Table 6 below.*

**Linear Regression**

In *Tables* *3*, *4*, and *5*, we compare the treatment effect of the gain and loss frames, experience and fact frames, and expert and non-expert frame to the control condition (no framing), respectively. Each treatment effect fails to meet the 95 percent standard of significance in each of the four vignettes. If our treatments do not explain the variation in participant responses, what does? We regressed our demographic variables step-wise with the responses for each vignette to examine whether any other variable can explain variation in attitudes.

Respondents appear to be pre-treated by information sources and identity characteristics. Consuming Fox and NBC news sources are significant for all vignettes apart from Fox under the self-quarantine vignette. Ideology follows expected pretreatment partisan and ideological information source tendencies, with a pattern of conservatives being more likely to disagree with self-quarantine, releasing detainees, and postponing NCAA football, but more likely to agree with continuing in-person only elections. Similar general patterns are displayed within group comparisons, when groups are strongly associated with party and ideology. Black respondents are more likely than White respondents to agree with releasing detainees and postponing NCAA football, but disagree with in-person only ballots. Men are more likely to disagree with self-quarantine and postponing NCAA football, but agree with in-person only elections. And finally, older respondents tend to disagree with releasing detainees and postponing NCAA football, but agree with in-person only ballots.

One deviation from this trend in similar responses across ideology and ideologically aligned groups is age within the self-quarantine vignette. Perhaps responding to higher personal risk, older respondents are more likely to agree with the self-quarantine vignette. Also, surprisingly, party affiliation does not have a significant effect across all conditions. This may indicate a genuine lack of relationship or limited relationship between party and agreement with the vignette. However, given the large number of independent variables investigated here, this may instead be an instance of multicollinearity.

In *Table 3*, below, we focus on the effects of gain and loss frames. Gain frame and loss frame are binary variables indicating if the respondents received this framing treatment. The reference group is the control group where respondents receive no treatment. Neither gain nor loss frames have a significant effect on agreement with the vignettes.

***Table 3: Gain vs. Loss Frame***

|  | **Self-Quarantine** | **Release Detainees** | **NCAA Football** | **In-Person Ballots** |
| --- | --- | --- | --- | --- |
| Gain Frame | 0.034 | 0.125 | -0.178 | 0.177 |
|  | (0.077) | (0.094) | (0.092) | (0.103) |
| Loss Frame | -0.008 | 0.136 | -0.125 | 0.069 |
|  | (0.077) | (0.094) | (0.093) | (0.103) |
| Ideology | -0.120^***^ | -0.295^***^ | -0.180^***^ | 0.198^***^ |
|  | (0.015) | (0.019) | (0.019) | (0.021) |
| Independent | -0.498^**^ | -0.167 | -0.093 | -0.377 |
|  | (0.160) | (0.195) | (0.192) | (0.213) |
| Unknown | 0.382^*^ | 0.135 | 0.038 | 1.094^***^ |
|  | (0.167) | (0.203) | (0.200) | (0.223) |
| Republican | 0.019 | -0.088 | -0.169^**^ | -0.157^*^ |
|  | (0.054) | (0.066) | (0.065) | (0.072) |
| Black | -0.004 | 0.680^***^ | 0.306^***^ | 0.478^***^ |
|  | (0.076) | (0.093) | (0.091) | (0.101) |
| Hispanic | 0.042 | 0.381^*^ | 0.690^***^ | -0.027 |
|  | (0.150) | (0.183) | (0.180) | (0.201) |
| Asian/Hawaiian | -0.379 | -0.574^*^ | 0.488 | -0.593 |
|  | (0.239) | (0.292) | (0.287) | (0.320) |
| Native American | 0.010  (0.252) | 0.345  (0.308) | 0.003  (0.303) | -0.224  (0.337) |
| Middle Eastern | 0.157  (0.296) | -0.238  (0.361) | 0.711^*^  (0.355) | -0.182  (0.395) |
| Other Ethnicities | -0.081  (0.078) | 0.168  (0.095) | 0.356^***^  (0.093) | 0.094  (0.104) |
| Female | 0.306^***^  (0.047) | -0.107  (0.057) | 0.206^***^  (0.056) | -0.335^***^  (0.062) |
| Education | 0.000 | 0.000 | 0.000 | 0.000 |
|  | (0.000) | (0.000) | (0.000) | (0.000) |
| Age | 0.003^*^ | -0.011^***^ | -0.004^*^ | -0.009^***^ |
|  | (0.001) | (0.002) | (0.002) | (0.002) |
| Fox Viewer | -0.035 | -0.519^***^ | -0.573^***^ | 0.553^***^ |
|  | (0.063) | (0.076) | (0.075) | (0.083) |
| NBC Viewer | 0.403^***^ | 0.333^***^ | 0.175^*^ | -0.547^***^ |
|  | (0.072) | (0.088) | (0.087) | (0.097) |
| CNN Viewer | 0.203^***^ | 0.244^**^ | -0.219^**^ | -0.352^***^ |
|  | (0.061) | (0.074) | (0.073) | (0.081) |
| ABC Viewer | 0.178^*^ | -0.029 | 0.105 | -0.191 |
|  | (0.087) | (0.106) | (0.104) | (0.116) |
| Facebook User | 0.124 | 0.289^**^ | 0.163 | 0.455^***^ |
|  | (0.083) | (0.101) | (0.099) | (0.111) |
| Twitter User | 0.247 | 0.217 | 0.338 | -0.052 |
|  | (0.148) | (0.180) | (0.178) | (0.198) |
| Instagram User | -0.254 | 0.111 | 0.074 | -0.265 |
|  | (0.140) | (0.170) | (0.168) | (0.186) |
| Constant | 5.821^***^ | 5.707^***^ | 5.706^***^ | 3.783^***^ |
|  | (0.117) | (0.142) | (0.140) | (0.156) |
| N | 4,214 | 4,214 | 4,214 | 4,214 |

| *Note:* | ^*^p<0.05^**^p<0.01^***^p<0.001 |
| --- | --- |

In *Table 4*, below, we focus on the effects of experience and fact frames. Experience frame and fact frame are binary variables indicating if the respondents received this framing treatment. The reference group is the control group where respondents receive no treatment. Neither experience nor fact frames have a significant effect on agreement with the vignettes.

***Table 4: Experience vs. Fact Frame***

|  | **Self-Quarantine** | **Release Detainees** | **NCAA Football** | **In-Person Ballots** |
| --- | --- | --- | --- | --- |
| Experience Fr. | 0.019 | 0.150 | -0.120 | 0.120 |
|  | (0.077) | (0.094) | (0.092) | (0.103) |
| Fact Frame | 0.006 | 0.111 | -0.185^*^ | 0.127 |
|  | (0.077) | (0.094) | (0.092) | (0.103) |
| Ideology | -0.120^***^ | -0.295^***^ | -0.179^***^ | 0.198^***^ |
|  | (0.015) | (0.019) | (0.019) | (0.021) |
| Independent | -0.498^**^ | -0.167 | -0.093 | -0.377 |
|  | (0.160) | (0.195) | (0.192) | (0.213) |
| Unknown | 0.384^*^ | 0.136 | 0.037 | 1.100^***^ |
|  | (0.167) | (0.203) | (0.200) | (0.222) |
| Republican | 0.022 | -0.088 | -0.170^**^ | -0.153^*^ |
|  | (0.054) | (0.066) | (0.065) | (0.072) |
| Black | -0.004 | 0.679^***^ | 0.304^***^ | 0.479^***^ |
|  | (0.076) | (0.093) | (0.091) | (0.101) |
| Hispanic | 0.041 | 0.380^*^ | 0.689^***^ | -0.029 |
|  | (0.150) | (0.183) | (0.180) | (0.201) |
| Asian/Hawaiian | -0.382 | -0.573^*^ | 0.493 | -0.600 |
|  | (0.239) | (0.292) | (0.287) | (0.320) |
| Native American | 0.013  (0.252) | 0.344  (0.308) | 0.001  (0.303) | -0.218  (0.337) |
| Middle Eastern | 0.156  (0.296) | -0.238  (0.360) | 0.711^*^  (0.355) | -0.182  (0.395) |
| Other Ethnicities | -0.082  (0.078) | 0.168  (0.095) | 0.358^***^  (0.093) | 0.090  (0.104) |
| Female | 0.306^***^  (0.047) | -0.106  (0.057) | 0.209^***^  (0.056) | -0.337^***^  (0.062) |
| Education | 0.000 | 0.000 | 0.000 | 0.000 |
|  | (0.001) | (0.000) | (0.000) | (0.000) |
| Age | 0.003^*^ | -0.011^***^ | -0.004^*^ | -0.009^***^ |
|  | (0.001) | (0.002) | (0.002) | (0.002) |
| Fox Viewer | 0.035 | -0.520^***^ | -0.574^***^ | 0.553^***^ |
|  | (0.063) | (0.076) | (0.075) | (0.083) |
| NBC Viewer | 0.404^***^ | 0.332^***^ | 0.171^*^ | -0.543^***^ |
|  | (0.072) | (0.088) | (0.087) | (0.097) |
| CNN Viewer | 0.203^***^ | 0.244^**^ | 0.219^**^ | -0.353^***^ |
|  | (0.061) | (0.074) | (0.073) | (0.081) |
| ABC Viewer | 0.179^*^ | -0.030 | 0.103 | -0.188 |
|  | (0.087) | (0.106) | (0.104) | (0.116) |
| Facebook User | 0.125 | 0.289^**^ | 0.162 | 0.457^***^ |
|  | (0.083) | (0.101) | (0.099) | (0.111) |
| Twitter User | 0.248 | 0.216 | 0.336 | -0.049 |
|  | (0.148) | (0.180) | (0.178) | (0.198) |
| Instagram User | -0.255 | 0.111 | 0.074 | -0.268 |
|  | (0.140) | (0.170) | (0.168) | (0.186) |
| Constant | 5.822^***^ | 5.707^***^ | 5.706^***^ | 3.785^***^ |
|  | (0.117) | (0.142) | (0.140) | (0.156) |
| N | 4,214 | 4,214 | 4,214 | 4,214 |

| *Note:* | ^*^p<0.05^**^p<0.01^***^p<0.001 |
| --- | --- |

In *Table 5*, below, we focus on the effects of expert and non-expert source cues. Expert frame and non-expert frame are binary variables indicating if the respondents received this source cue treatment. The reference group is the control group where respondents receive no treatment. Neither expert nor non-expert source cues have a significant effect on agreement with the vignettes.

***Table 5: Expert vs. Non-Expert Frame***

|  | **Self-Quarantine** | **Release Detainees** | **NCAA Football** | **In-Person Ballots** |
| --- | --- | --- | --- | --- |
| Expert Frame | 0.013 | 0.111 | -0.176 | 0.083 |
|  | (0.077) | (0.094) | (0.093) | (0.103) |
| Non-Expert Fr. | 0.013 | 0.150 | -0.128 | 0.163 |
|  | (0.077) | (0.094) | (0.092) | (0.103) |
| Ideology | -0.120^***^ | -0.295^***^ | -0.179^***^ | 0.198^***^ |
|  | (0.015) | (0.019) | (0.019) | (0.021) |
| Independent | -0.498^**^ | -0.167 | -0.093 | -0.377 |
|  | (0.160) | (0.195) | (0.192) | (0.213) |
| Unknown | 0.384^*^ | 0.135 | 0.036 | 1.101^***^ |
|  | (0.167) | (0.203) | (0.200) | (0.223) |
| Republican | 0.021 | -0.089 | -0.172^**^ | -0.154^*^ |
|  | (0.054) | (0.066) | (0.065) | (0.072) |
| Black | -0.004 | 0.680^***^ | 0.305^***^ | 0.479^***^ |
|  | (0.076) | (0.093) | (0.091) | (0.101) |
| Hispanic | 0.041 | 0.385^*^ | 0.695^***^ | -0.022 |
|  | (0.150) | (0.183) | (0.180) | (0.201) |
| Asian/Hawaiian | -0.382 | -0.571 | 0.494 | -0.595 |
|  | (0.240) | (0.292) | (0.287) | (0.320) |
| Native American | 0.013  (0.252) | 0.345  (0.308) | 0.002  (0.303) | -0.216  (0.337) |
| Middle Eastern | 0.156  (0.296) | -0.241  (0.361) | 0.708^*^  (0.355) | -0.187  (0.395) |
| Other Ethnicities | -0.082  (0.078) | -0.168  (0.095) | 0.358^***^  (0.093) | 0.090  (0.104) |
| Female | 0.305^***^  (0.047) | -0.108  (0.057) | 0.205^***^  (0.056) | -0.339^***^  (0.062) |
| Education | 0.000 | 0.000 | 0.000 | 0.000 |
|  | (0.000) | (0.000) | (0.000) | (0.000) |
| Age | 0.003^*^ | -0.011^***^ | -0.004^*^ | -0.009^***^ |
|  | (0.001) | (0.002) | (0.002) | (0.002) |
| Fox Viewer | 0.035 | -0.520^***^ | -0.574^***^ | 0.551^***^ |
|  | (0.063) | (0.076) | (0.075) | (0.083) |
| NBC Viewer | 0.404^***^ | 0.332^***^ | 0.173^*^ | -0.544^***^ |
|  | (0.072) | (0.088) | (0.087) | (0.083) |
| CNN Viewer | 0.203^***^ | 0.245^**^ | 0.220^**^ | -0.350^***^ |
|  | (0.061) | (0.074) | (0.073) | (0.081) |
| ABC Viewer | 0.179^*^ | -0.031 | 0.103 | -0.191 |
|  | (0.087) | (0.106) | (0.104) | (0.116) |
| Facebook User | 0.125 | 0.288^**^ | 0.161 | 0.455^***^ |
|  | (0.083) | (0.101) | (0.099) | (0.111) |
| Twitter User | 0.248 | 0.219 | 0.339 | -0.044 |
|  | (0.148) | (0.181) | (0.178) | (0.198) |
| Instagram User | -0.255 | 0.112 | 0.076 | -0.266 |
|  | (0.140) | (0.170) | (0.168) | (0.186) |
| Constant | 5.822^***^ | 5.708^***^ | 5.707^***^ | 3.787^***^ |
|  | (0.117) | (0.142) | (0.140) | (0.156) |
| N | 4,214 | 4,214 | 4,214 | 4,214 |

| *Note:* | ^*^p<0.05^**^p<0.01^***^p<0.001 |
| --- | --- |

Here we also provide some explanation of the coding of the remaining independent variables in *Tables 3*, *4*, and *5*. Ideology is coded from 1 to 7 representing “very liberal,” “liberal,” “slightly liberal,” “moderate, middle of the road,” “slightly conservative,” “conservative,” and “very conservative,” respectively. Party identification is created from a 7-point scale with a “don’t know” category—it is recoded into a categorical variable with 4 categories. Democrat is the reference category to Republican, Independent, and unknown. This last category consists of “don’t know” responses. Ethnicity is also a categorical variable, using White as the reference to the displayed Black, Hispanic, Asian/Hawaiian, Native American, Middle Eastern and Other Ethnicities. Female is the reference category for gender to the shown male and other gender. Education is coded from 1 to 7 representing “No high school,” “High school graduate or G.E.D.,” “Some college but no degree,” “Associate degree,” “Bachelor's degree,” “Post-graduate degree” and “Professional degree,” respectively. Age is the respondent’s age in years. Finally, respondents were given an open-ended question regarding the media sources that they follow. The news and social media variables are binary variables which indicates if respondents identified Fox, NCB, CNN, Facebook, Twitter, or Instagram in response to this query.

Lastly, *Table 6*, below, provides the simplified model used as the basis for *Figure 3* in the main text of the paper. The coefficients in the table are not standardized as those in corresponding figure. This model separates treatments according to the three main treatment groups and the control group. Treatment, then, is a dummy variable with control groups as the reference category, expertise is a dummy variable with nonexperts and the control group as the reference, gain frame is a dummy variable with loss frame and the control group as the reference, and fact frame is a dummy variable with experience frame and the control group as the reference. Changing the reference categories does not change our main findings (or lack thereof).

Party ID is coded from –3 to 3 representing “strong Democrats,” “weak Democrats,” “independent but lead Democrat,” “completely independent,” “independent but lean Republican,” “weak Republican,” and “strong Republicans,” respectively. Many respondents (N=955) replied “Don’t Know.” These individuals were coded as zero along with independents within the Party ID variable and a separate dummy variable (DK PID) is added to distinguish these “Don’t Know” respondents from self-identifying independents and partisans. Ideology is coded from 1 to 7 representing “very liberal,” “liberal,” “slightly liberal,” “moderate, middle of the road,” “slightly conservative,” “conservative” and “very conservative,” respectively. Age is the respondent’s age in years. Fox and NBC are each dummy variables that indicate if the respondent identified any Fox or NBC new outlets respectively as sources of news they regularly consume.

***Table 6: Simplified OLS Model (Associated with Figure 3 in Main Text)***

|  | **Self-**  **Quarantine** | **Release Detainees** | **NCAA  Football** | **In-Person Ballots** |
| --- | --- | --- | --- | --- |

| Treatment | -0.004 | 0.145 | -0.082 | 0.101 |
| --- | --- | --- | --- | --- |
|  | (0.084) | (0.099) | (0.097) | (0.113) |
| Expertise | -0.002 | - | - | -0.085 |
|  | (0.049) | - | - | (0.066) |
| Gain Frame | 0.047 | -0.002 | -0.056 | 0.110 |
|  | (0.049) | (0.060) | (0.059) | (0.066) |
| Fact Frame | -0.011 | -0.043 | -0.067 | 0.004 |
|  | (0.049) | (0.060) | (0.059) | (0.066) |
| Female | 0.299^***^ | -0.111 | 0.211^***^ | -0.340^***^ |
|  | (0.047) | (0.057) | (0.056) | (0.062) |
| Education | 0.000 | 0.000 | 0.000 | 0.000 |
|  | (0.000) | (0.000) | (0.000) | (0.000) |
| Party ID | -0.011 | -0.069^***^ | -0.063^***^ | -0.023 |
|  | (0.012) | (0.015) | (0.014) | (0.016) |
| Don’t Know PID | -0.109 | -0.065 | -0.031 | 0.764^***^ |
|  | (0.066) | (0.081) | (0.080) | (0.089) |
| Ideology | -0.116^***^ | -0.283^***^ | -0.170^***^ | 0.194^***^ |
|  | (0.016) | (0.019) | (0.019) | (0.021) |
| Age | 0.004^*^ | -0.013^***^ | -0.006^***^ | -0.010^***^ |
|  | (0.001) | (0.002) | (0.002) | (0.002) |
| Fox Viewer | 0.048  (0.063) | -0.525^***^  (0.077) | -0.577^***^  (0.075) | 0.544^***^  (0.084) |
| NBC Viewer | 0.409^***^  (0.072) | 0.306^***^  (0.089) | 0.152^*^  (0.087) | -0.557^***^  (0.097) |
| CNN Viewer | 0.207^***^ | 0.216^**^ | 0.223^**^ | -0.372^***^ |
|  | (0.061) | (0.075) | (0.073) | (0.081) |
| ABC Viewer | 0.178^*^ | -0.028 | 0.106 | -0.193 |
|  | (0.087) | (0.107) | (0.105) | (0.117) |
| Facebook User | 0.125 | 0.265^**^ | 0.169 | 0.432^***^ |
|  | (0.083) | (0.101) | (0.100) | (0.111) |
| Twitter User | 0.249 | 0.209 | 0.324 | -0.053 |
|  | (0.148) | (0.181) | (0.178) | (0.198) |
| Instagram User | -0.263 | 0.191 | 0.108 | -0.211 |
|  | (0.139) | (0.170) | (0.167) | (0.186) |
| Constant | 5.769^***^ | 5.837^***^ | 5.787^***^ | 3.830^***^ |
|  | (0.111) | (0.136) | (0.133) | (0.148) |
| Observations | 4,214 | 4,214 | 4,214 | 4,214 |
| *Note:* | ^*^p<0.05^**^p<0.01^***^p<0.001 | | | |

**APPENDIX D: Original Grant Proposal**

**To Believe or To Not Believe:**

**Reception of Expert Cues during COVID-19**

**Home Department/Unit:** Political Science

**Abstract:** Extant literature finds that Americans increasingly distrust experts. We build on this in the context of a global crisis, hypothesizing that expert recommendations aiming to mitigate the spread of COVID-19 are ineffective because people do not trust experts. We propose to test this in an online survey experiment that varies whether a source is expert or non-expert as well as whether the information given is fact or experience and positive or negative. Examining the reception of information from experts in this context can inform communication strategies that could save lives in large-scale crises.

**Research Strategy**

**Background:**

As the COVID-19 pandemic surged, news outlets were rife with stories about people ignoring social distancing recommendations. A Louisiana pastor defied the Governor's order against gatherings of more than 50 people by hosting over 1,000 churchgoers at a Sunday service. In New Jersey, 47 people organized a “Corona party.” Thousands of students continued to gather for Spring Break in Florida. This was despite overwhelming expert communication about the dangers of such gatherings. Given experts’ clear warnings, why do Americans continue to defy health and safety recommendations?

Research on the growing distrust of experts and rise of anti-intellectualism in America suggests one explanation (see Merkley 2019)—that these recommendations are ignored because people don’t trust experts. Indeed, survey research shows that trust in experts, and thus likelihood of believing experts, has declined over time (Archer and Ron-Levey 2020). We hypothesize that this declining trust in experts is driving people to ignore experts’ COVID-19 health and safety guidelines. In particular, we predict that because of the increasing distrust in experts, people will be less likely to follow public health guidelines when they originate from an expert source as compared to when they originate from a non-expert source—*even though* the experts, presumably, have more (and more reliable) information on COVID-19.

That the public often relies on cues—especially source cues—to process information is widely acknowledged. Because much of the public lacks background knowledge to properly interpret and critique new information, this is not necessarily a bad thing. Indeed, people still assess *source* credibility, including intelligence, integrity, and moral priorities (Hendriks et al. 2015; Darmofal 2005), even when they don’t assess *information* credibility. Yet people’s assessment of source credibility is likely shaped by today’s anti-intellectualism sentiment, leading people to be skeptical of information from experts—associating the experts with elite institutions, priming a perceived conflict between elites and ordinary citizens, and instead trusting practical knowledge or “common sense” (Merkley 2019). Further, expertise may also prime partisan identity. For example, Republicans have recently become skeptical of the scientific community, as Republican elites associate institutions of higher education with partisan out-groups and in doing so question their moral integrity (Motta 2018, Cramer 2016). This suggests that Republicans will be even *less* receptive to information from health experts.

Further, in the particular case of COVID-19, we have seen concerted efforts to delegitimize health officials and call into question their competence, reliability, and trustworthiness—suggesting the recent pandemic is indeed a context where anti-expert sentiment guides both attitudes and, importantly, behavior. Some media elites, for example, have directly challenged the “public health establishment.” On *Tucker Carlson Tonight,* the host stated: “One of the main lessons of this crisis is that the public health establishment failed us badly...We’re being asked to trust these same people without hesitation and for the most part we are doing that. In other words, the experts failed yet the experts now have more power than ever before. It’s bewildering” (Carlson 2020). On the other hand, though, a health pandemic could be a context in which people suspend their anti-expert sentiment—at least towards health workers—instead prioritizing their own safety. A recent Gallup poll, for example, found that while residents of both Western Europe and the U.S. have relatively low trust in government, their trust in medical workers nearly matches the rest of the world (Archer and Ron-Levey 2020).

Given the extremely high stakes in this crisis—including both human survival and economic safety—which depend *greatly* on individuals’ daily behaviors, it is of vital importance to understand how people integrate information from health experts into their beliefs and behavior. In particular, the growing skepticism of experts, as well as elites’ corresponding rhetoric both generally and in the context of COVID-19, leads us to believe that for many, expert opinion is downgraded in favor of “common sense”—something that could result in faster spreading of the virus and thus more deaths. In this project we examine how expert versus non-expert source cues matter in the context of the current COVID-19 health crisis, predicting that people will be more likely to believe statements made by non-experts than by experts, and that this will be moderated by both trust in experts and partisanship. Results would suggest a different public relations tactic in a health crisis than the one that currently predominates—one focused less on experts and more on average Americans.

**Significance:**

The COVID-19 pandemic highlights the importance of the public’s role in either mitigating or exacerbating the impact of large-scale crises, including—but not limited to—the current health crisis. If the public does not trust information from experts, they are less likely to conform to guidelines and recommendations that would limit the negative impacts of disasters and thus benefit the community. In cases such as COVID-19, this poor information quality has very tangible negative outcomes as it can lead to destructive behavior such as refusing to socially distance oneself. Thus, understanding the dynamics of the dissemination of expert information during precarious times such as these is of fundamental importance. Our project in particular can help public officials build more constructive communication strategies, which would enhance public safety during not only this crisis, but also similar large-scale crises.

**Innovation:**

While much research on national crises explores potential policy solutions, executive orders, bureaucratic powers, and the like, this project seeks to fill in a gap in better understanding *individual* behavior during national crises. Incorporating existing theories from political psychology, including the effects of source cues on attitudes and behaviors, we investigate a unique aspect of the COVID-19 crisis—by examining the public’s reception of the information they receive from medical experts, our research can test whether a communication strategy that focuses on expert opinion is subpar in mitigating the spreading of the virus. In doing so, we also improve upon previous social science research on source cues in two ways. First, we directly test how individuals incorporate information from experts *as compared to* average Americans in a situation in which experts have better and more complete information. Second, we examine this in the context of a crisis where the stakes for ignoring expert opinion are quite high. While extant research indeed suggests the growing distrust of experts (Merkley 2019, 2020), examining whether or not this distrust remains in a high-stakes context such as the COVID-19 pandemic can tell us how extreme and thus how damaging this trend is. Conversely, if we instead find that during this health crisis people are receptive to information from experts, this would suggest that expert distrust is conditional on costs and that a communication strategy that relies on expert opinion is, in fact, a constructive strategy during such a large-scale crisis.

**Research Design:**

We hypothesize that Americans are more likely to believe information about COVID-19 when received from an average person rather than if that *same* information is received from an expert. To test this, we conduct a survey experiment that varies both source cues and type of information given, as this could influence how people incorporate the information from different sources. For all respondents, we first measure the following possible moderators: partisanship, ideology, age, gender, income, education, political knowledge, media exposure, religion, and geographic location. Participants are then randomly assigned to 1 of the 9 conditions (8 treatment and 1 control group), and the treatments vary whether the source is an expert or non-expert, whether the information given is fact or experience, and whether this information is positive or negative (see *Table 1*). Below is an example of one of our treatments:

All Groups: As states have enacted shelter in place orders and shutdowns of all nonessential businesses, the impact on religious institutions varies from state to state. Some states have left the decision to host congregations up to the religious institutions while others have considered religious congregations nonessential and therefore ordered them closed. [*Groups 1-4:* Dr. Kane, an epidemiologist (epidemiology: the study of health and disease), / *Groups 5-8:* Mr. Kane, a congregation member,] says: [*Groups 1 + 5:* “The COVID-19 virus exists in the surrounding for hours. Even sparsely attended congregations with spaced seating expose attendees to an unnecessary risk of spreading or contracting the virus. All states should consider religious institutions nonessential and force the closure of in-person congregations.” / *Groups 3 + 7:* “There are a number of stories of religious organizations hosting smaller services, following social distancing guidelines and disinfection practices, but still seeing many in-person attendees getting sick or testing positive for COVID-19. All states should consider religious institutions nonessential and force the closure of in-person congregations.” / *Groups 2 + 6:* “Freedom of Conscience is a bedrock of American society, and religious organizations that are following health guidelines offer religious services to people and do not increase the risk of contamination any more than going to the grocery store. All states should allow religious institutions to decide whether to host congregations or close for the time being.” / *Groups 4 + 8:* “Freedom of Conscience is a bedrock of American society, and I am familiar with several organizations that are following health guidelines while offering religious services to people and are not experiencing outbreaks within their congregations. All states should allow religious institutions to decide whether to host congregations or close for the time being.”]^^[[1]](#footnote-1)^^

Respondents will read three other analogous statements within the same condition—the three other statements will examine different types of experts and issues in order to gauge a more comprehensive estimation of the reception of expert cues in the COVID-19 crisis. Our dependent variable is the incorporation of the information received—we measure this by asking participants their agreement with the information given. For example, for the treatment above participants are asked: “How much do you agree with the statements that all states should order the closure of religious institutions?” with response options from 1 (strongly agree) to 7 (strongly disagree). After the dependent variable is measured, we measure our second hypothesized moderator—trust in source cue—by asking participants: “How much do you trust [source]?” with response options from 1 (completely) to 7 (not at all). This is asked *post*-treatment so as to not prime participants. At the end of the survey, all respondents are debriefed and told that the information provided in the survey was fictional.

***Table 1: Assignment of Treatment Groups***

| **Source Cue** | **Information** | | | | **Control Group** |
| --- | --- | --- | --- | --- | --- |
|  | **Fact** (statement explains support or opposition using verifiable facts) | | **Experience** (statement explains support or opposition using personal experience) | | Group 9 (N~500) |
|  | **Positive** (statement supports the issue) | **Negative** (statement opposes the issue) | **Positive** (statement supports the issue) | **Negative** (statement opposes the issue) |  |
| **Expert** (statement made by an expert with relevant qualifications) | Group 1 (N~500) | Group 2 (N~500) | Group 3 (N~500) | Group 4 (N~500) |  |
| **Non-Expert** (statement made by a non-expert with no relevant qualifications) | Group 5 (N~500) | Group 6 (N~500) | Group 7 (N~500) | Group 8 (N~500) |  |

**Data Collection and Sharing:**

The survey will be built using Qualtrics and run with survey participants from Lucid, an online survey platform. Due to the high number of users on Lucid, once deployed, data collection will take no longer than one week. This entire process—from the creation of the survey to the submission of the paper—can be done online. Thus, no physical access to university resources is required. We also do not store any personally identifiable data from the respondents. The unidentifiable data will be stored on a personal computer and released when the results of the experiment are published. The results from this experiment will be useful not just in this particular study, but also for future avenues of research, including an analysis of whether the reception of information from experts changes when the COVID-19 crisis is over, an examination of competing cues from experts and non-experts, and a study on media framing of expert cues.

**References:**

Archer, Kristjan and Ilana Ron-Levey. 2020. “Trust in Government Lacking on COVID-19's Frontlines.” *Gallup.* https://news.gallup.com/opinion/gallup/296594/trust-government-lacking-frontlines-covid.aspx.

Carlson*,* Tucker*.* “Tucker Carlson Tonight". Season 5 Episode 67. *Fox* *Broadcasting*. April 2, 2020.

Cramer, Katherine J. 2016. *The Politics of Resentment: Rural Consciousness in Wisconsin and the Rise of Scott Walker*. University of Chicago Press, 2016.

Darmofal, David. 2005. "Elite Cues and Citizen Disagreement with Expert Opinion." *Political Research Quarterly* 58(3): 381-395.

Hendriks, Friederike, Dorothe Kienhues, and Rainer Bromme. 2015. "Measuring Laypeople’s Trust in Experts in a Digital Age: The Muenster Epistemic Trustworthiness Inventory (METI)." *PloS One* 10(10): 1-20.

Merkley, Eric. 2020. "Anti-Intellectualism, Populism, and Motivated Resistance to Expert Consensus." *Political Research Quarterly.* Forthcoming.

Merkley, Eric. 2019. "When Experts Talk, Does Anyone Listen? Essays on the Limits of Expert Influence on Public Opinion." PhD diss., University of British Columbia.

Motta, Matthew. 2018. "The Dynamics and Political Implications of Anti-Intellectualism in the United States." *American Politics Research* 46(3): 465-498.

**APPENDIX E: Reporting Standards**

1. **Hypotheses**

- Hypotheses are laid out in the main text, under the “Theory” section.

1. **Subjects and Context**
   - Those eligible to take the study had to be in the United States and over age 18.
   - These eligibility criteria were selected by the researchers but conducted by Lucid (https://luc.id/).
   - The study began on May 20^th^, 2020 and ran until we had enough participants (4,500).
   - All of this was conducted online.
2. **Allocation Method**
   - Subjects were randomly assigned to treatments at the individual level.
   - See Appendix B, *Table 1* for balance checks on demographic characteristics
3. **Treatments**
   - Treatment text can be found in Appendix A.
   - Treatments were delivered online using the Qualtrics survey platform

(https://www.qualtrics.com/).

- - Deception was used, but participants were debriefed:

*Thank you for your participation in this experiment. The goal of this study was to determine whether people are more likely to believe statements made by ordinary American citizens or statements made by experts when considering behaviors and policy preferences in the context of COVID-19. We think that the source of a statement influences people's trust of that information. Statements and sources provided in this experiment are completely fictional.*

*Your participation is not only appreciated by the researchers involved, but the data collected will help us understand communication dynamics for information dissemination during the outbreak. A closer examination of the sources Americans trust most can enhance public safety both now and in the future.*

*If you have any questions about this study, please contact Christopher Howell at ch57@email.sc.edu or Dr. Elizabeth Connors at CONNORS4@mailbox.sc.edu).*

*Finally, we urge you not to discuss this study with anyone else who is currently participating or might participate later. As you can understand, we will not be able to examine the effect of cues in participants who know about the true purpose of the project beforehand.*

- - Manipulation checks were conducted (see Appendix A).

1. **Results**
   - **Outcome Measures and Covariates**
     - The questionnaire that we administered to create variable measures can be found in Appendix A.
     - Analysis of treatment effects was preregistered in grant proposal (Appendix D), but observational analyses were exploratory.
   - **CONSORT:**
     - 4,722 (of all eligible participants) joined the study, leading to a breakdown of participants to groups as follows: 1= 498; 2=503; 3=495; 4=536; 5=518; 6=507; 7=540; 8=559; 9=554.
     - 36 participants did not respond to the dependent variable measure (by group: 1=3; 2=0; 3=1; 4=2; 5=4; 6=6; 7=3; 8=2; 9=3).
     - This left us with 4,686 participants, with the following breakdown for statistical analysis by group: 1=495; 2=503; 3=494; 4=534; 5=514; 6=501; 7=537; 8=557; 9=551.
   - **Statistical Analysis**
     - Statistical analysis and defense of that analysis can be found in the main text, under the “Results” section.
2. **Other Information**
   - This experiment was reviewed and approved by the University of South Carolina’s IRB (Pro00099931).
   - The experimental protocol was preregistered in grant application (see Appendix D).
   - The source of funding was an internal grant from the University of South Carolina (see Appendix D), who had no role in the research other than the funding of it (i.e., there were no restrictions or arrangements regarding what findings could be published).
   - Replication data and code are available at the Journal of Experimental Political Science Dataverse within the Harvard Dataverse Network, at: doi: 10.7910/DVN/0Q1F1U.

1. Note that with the quickly changing dynamics of this crisis, treatments may need to be slightly updated by the time the study is run. [↑](#footnote-ref-1)
